# The Resilience Responsibility Gap: how institutions distribute the burden of adaptation under AI-driven structural disruption

**DOI:** 10.3389/fpsyg.2026.1974636

**Published:** 2026-10-16

**Authors:** Bora Basaran

**Affiliations:** 1 Anadolu University, Eskişehir, Türkiye; 2 Department of Foreign Languages Education, Faculty of Education, Anadolu University, Eskisehir, Türkiye

**Keywords:** artificial intelligence, organizational adaptation, psychological capital, reskilling, structural disruption, worker participation, workforce resilience

## Abstract

**Introduction:**

AI-drivenworkforce disruption is structural rather than episodic and is reshaping skills, tasks, job roles, career prospects and job stability. Resilience research has traditionally focused on individuals, yet adaptation is required at the individual, organizational, institutional and societal levels. This study examines who is held responsible for adaptation in institutional documents and whether the demands placed on workers are matched by accessible resources.

**Methods:**

A structured, codebook-based comparative document analysis was conducted on 18 publications from the OECD (*n* = 9), Eurofound (*n* = 5), EU-OSHA (*n* = 2), the European Commission’s Joint Research Center (*n* = 1) and the International Labor Organization (*n* = 1). Published between 2021 and 2026, the documents were selected purposively, and the corpus is dominated by OECD and EU institutions. They were coded with a 28-field codebook, supported by a page-level audit trail, in two separate coding passes conducted by the author.

**Results:**

In this corpus, no document assigned workers primary responsibility for adaptation, and individual responsibility was never coded as central; both passes classified all 18 documents as non-individual-dominant. The distribution across the four balance categories is reported in the Results and should be interpreted cautiously, since the passes agreed on the exact category in 10 of 18 documents. A Resilience Responsibility Gap, defined as a mismatch between adaptation demands, resources and responsibility, was clear in seven documents, possible or limited in eight and absent in three. Five inductively identified subtypes concern access to skills, worker participation, psychosocial prevention, social protection and organizational capacity. Psychological Capital did not appear as an explicit construct in any document, although related resources such as self-efficacy, adaptability, coping and trust did.

**Discussion:**

Psychological capital was not measured here, so its structural conditioning is advanced as a theoretical proposition rather than a demonstrated finding: the association between psychological capital and sustainable adaptation is expected to be moderated by the organizational and institutional resource configuration, a proposition for future testing. Workforce resilience is accordingly conceptualized as distributed adaptive capacity, arising from the alignment between adaptation demands and the individual, organizational and institutional resources available to meet them.

## 1 Introduction

It is often argued that individuals themselves should manage the changes currently reshaping work as a result of globalization and automation (Fejes, 2010; Rice et al., 2022). They are expected to learn about AI, continually adapt, take control of their own lives, and remain mentally strong in the face of ongoing disruption. There is some evidence for this view: when a job involves uncertainty, individuals with greater career adaptability and a stronger reserve of personal psychological resources tend to fare better than those without them (Avey et al., 2011; Luthans and Youssef-Morgan, 2017; Rudolph et al., 2017). However, if resilience is expected to come entirely from the individual, attention shifts away from the reasons adaptation is necessary, the companies that decide to adopt new technologies, the ways in which they design jobs and train employees, their decisions to offer short-term rather than long-term contracts, and the legal rights and protections employees should have. In that case, there is no longer any need to consider who should pay for adaptation because the question has already been answered.

This arrangement becomes more difficult to sustain when disruption becomes a constant feature of working life. Occupational health research distinguishes acute stressors, from which people can recover, from chronic demands that deplete resources over time (Ganster and Rosen, 2013; Hobfoll et al., 2018), and task-based evidence indicates that AI-related change is continuing rather than one-off (Acemoglu and Restrepo, 2019; Felten et al., 2021; Eloundou et al., 2024). The development of AI changes the tasks employees perform, reduces their influence over how they work through algorithmic direction, supervision and evaluation, and alters the meaning of job security, individual choice and future prospects (Kellogg et al., 2020; Jarrahi et al., 2021; Parker and Grote, 2022). The question is therefore not only whether workers can adapt (Rudolph et al., 2017; Hartmann et al., 2020), but also whether the burdens created by continuous transformation are matched by sufficient resources and who should provide them.

Institutional documents are where this issue is most clearly revealed. Documents produced by the OECD, ILO, Eurofound, and EU-OSHA, including reports, briefs, guidelines, review articles, and handbooks, do more than describe how labor markets have evolved. They also perform a specific normative function: they identify who is responsible for adaptation, define what constitutes an appropriate organizational response, indicate where resources (primarily from the public sphere) are expected to come from, and specify the competences workers should develop over time. In policy-analytical terms, these documents also construct a representation of the problem that makes the issue understandable while identifying where a possible solution may lie (Bacchi, 2012; Entman, 1993; van Hulst and Yanow, 2016). Reading these documents comparatively allows the uneven distribution of the burden of adaptation to be examined rather than accepted as a given. AI-driven disruption is understood here as part of a broader process of digital transformation. The corpus therefore also includes documents on algorithmic management, robotics and digitalization in which AI is one of several technologies, and the analysis refers to AI-driven and digitally mediated disruption where this broader scope matters. A critical analysis of these documents might initially be expected to reveal particular language games through which responsibility for adaptation is transferred to workers under the guise of empowerment through the discourses of employability and resilience (Fejes, 2010; Joseph, 2013; Gill and Orgad, 2018; Rice et al., 2022; Dobbins and Plows, 2023). Rather than assuming that institutional documents transfer responsibility to workers, the present study examines empirically how responsibility is allocated and treats documents in which responsibility is not individualized as analytically informative rather than as anomalies.

Institutional labor and skills discourse has been examined before. Analyses of employability and lifelong learning policy have shown how responsibility for employment risks is constructed as a matter of individual self-management (Fejes, 2010; Mertanen et al., 2020; Rice et al., 2022), and an analysis of 195 future-of-work policy documents published between 2013 and 2018 identified re- and upskilling as the dominant policy answer to technological change (Schlogl et al., 2021). These studies, however, focus on skills and employability discourse, largely predate the diffusion of generative AI, and do not examine how responsibility is allocated across individual, organizational and policy actors, or whether the demands placed on workers are matched by accessible resources. The present study addresses this more specific gap.

The central concept of this study is the Resilience Responsibility Gap: a mismatch between the role workers are expected to play in adapting to structural change and the resources, protections and rights available to them. The concept is intentionally narrow in scope. It does not simply indicate that stress, uncertainty, and new skill requirements are widespread, as these are universal. Instead, it describes a specific structural condition in which substantial pressures to adapt arise alongside limited access to reskilling, employer-supported learning that takes time constraints into account, opportunities for job redesign, psychological safety, worker input, meaningful control over work, clear rules governing the use of AI, informal discussion among stakeholders, social benefits, support with job transitions, or fair access to any of these resources. Framed in this way, the gap is not assumed to be inevitable: it is an analytical category that can also identify configurations in which demands and resources are aligned. This paper makes three contributions. Empirically, it provides a codebook-based, page-referenced comparative analysis of how 18 documents from OECD- and EU-centered institutions allocate responsibility for adaptation across individual, organizational and policy levels, and it documents that Psychological Capital is absent as an explicit construct even though its components appear. Conceptually, it introduces the Resilience Responsibility Gap as a relational construct linking adaptation demands, resources and responsibility. Theoretically, it proposes to conceptualize workforce resilience as distributed adaptive capacity. This proposal builds on multilevel resilience research and on COR’s resource caravan passageways, and adds an explicit account of who is responsible for providing resources, an institutional level of analysis and a distributional dimension (Section 5.1). Four research questions are addressed:

*RQ1*: How is AI-driven and digitally mediated structural workforce disruption framed in major institutional labor and employment documents?

*RQ2*: How is responsibility for adapting to structural disruption distributed across individual workers, organizations/employers, and policy/system actors?

*RQ3*: What adaptation resources, including reskilling, organizational support, worker participation, psychosocial resources and policy protection, are positioned as enabling sustainable workforce adaptation?

*RQ4*: Where do mismatches emerge between adaptation demands and the resources or institutional responsibilities available to meet them?

Building on these questions, the study pursues an integrative theoretical aim: to examine what the observed patterns imply for conceptualizing workforce resilience as an individual, organizational and institutional phenomenon. This aim is not addressed through coding but through interpretive synthesis of the findings in the Discussion (Section 5.1).

## 2 Theoretical background

### 2.1 From episodic stress to structural workforce disruption

In occupational health psychology, a distinction was traditionally made between acute and chronic stressors, with chronic stressors shown to involve different mechanisms and operate over different timelines than single, discrete events (Ganster and Rosen, 2013; Epel et al., 2018). Structural change in the workforce does not fit comfortably into either category. Instead, it may be better understood as altering the boundary conditions that define a job: What does the job involve, how is performance measured, how much flexibility is built into the way it is performed, what opportunities exist for professional development and promotion, and how stable is the position?

The structural change is cumulative and open-ended. Task-based analyses describe people and AI systems exchanging activities across jobs rather than AI replacing entire jobs (Acemoglu and Restrepo, 2019; Felten et al., 2021; Eloundou et al., 2024), and evidence from an online labor market indicates that the release of generative AI tools was followed within months by fewer jobs and lower earnings for freelancers in exposed occupations (Hui et al., 2024). Because new capabilities continue to be introduced, adaptation is required repeatedly rather than once. Second, the impact is socio-technical and institutional rather than purely technological. Whether AI supports autonomy or increases workload and distress depends on how it is integrated into work (Parker and Grote, 2022; Makarius et al., 2020). The same technology can have different consequences depending on the job and organization into which it is introduced. Electronic performance monitoring, for example, has different effects depending on whether it is designed to support employees or to conduct surveillance (Kellogg et al., 2020; Jarrahi et al., 2021; Siegel et al., 2021; Benlian et al., 2022; Vignola et al., 2023; Bowdler et al., 2026).

And finally the structural change redistributes exposure rather than simply increasing it. Exposure varies according to occupational position, skill level, contract type, company size, and age (Felten et al., 2021; Rani and Furrer, 2021). Access to the resources needed to manage structural change also varies according to these characteristics. Structural disruption must therefore be understood as a distributional issue, which is why individual-level coping alone provides an insufficient explanation of its broader significance.

### 2.2 Workforce resilience and psychological capital

Hope, self-efficacy, resilience, and optimism together form Psychological Capital (PsyCap) (Luthans et al., 2007). Within the PsyCap model, it is understood as a higher-order, state-like resource. Empirical measures of the construct include the Psychological Capital Questionnaire. Meta-analytical evidence shows that PsyCap is positively associated with attitudes, behaviors, and performance (Avey et al., 2011; Loghman et al., 2023). It has also been recognized as an evidence-based approach within positive organizational behavior research (Luthans and Youssef-Morgan, 2017). Despite criticisms concerning its higher-order measurement, PsyCap remains one of the key tools for understanding adaptability and resilience at work (Dawkins et al., 2013).

Two qualifications are important. First, workplace resilience is not simply an individual characteristic; it is a multilevel issue. Reviewers have distinguished among individual, team, and organizational resilience, while evidence indicates that employee resilience is strongly shaped by the organization rather than being an inherent disposition (Hartmann et al., 2020; Kuntz et al., 2017; Näswall et al., 2019). Evidence from teaching workforces points in the same direction: shared instructional leadership is associated with teacher job satisfaction through teacher self-efficacy and professional resilience (Dilekçi et al., 2026), and psychological capital predicts teachers’ professional resilience capacities (Kavrayıcı et al., 2024). Career adaptability has a similar multilevel quality: adaptivity, adapting responses, and adaptation outcomes are not necessarily equivalent and vary according to contextual conditions, such as how well a person’s adapting response matches the challenges of their career environment (Savickas and Porfeli, 2012; Rudolph et al., 2017; Kvasková et al., 2023). Psychological safety is another feature of the work environment that enables individuals to act courageously rather than a characteristic possessed by an individual person (Edmondson, 1999; Newman et al., 2017; Edmondson and Bransby, 2023).

The politics surrounding resilience discourse is the second reason why this language raises suspicion. Critical scholarship, for example, shows that discussions of resilience and employability have helped shift responsibility for career-related risks toward individuals (Joseph, 2013; Gill and Orgad, 2018; Mertanen et al., 2020; Suslovic and Lett, 2024), while leaving the structures that produce those risks unaddressed (Brown, 2015). Vincent et al. (2024) describe a related process in the regulation of diversity management as relational responsibilisation, in which responsibility is dispersed across several actors in ways that can leave obligations unevenly met; this study adapts the concept to the allocation of responsibility for workforce adaptation. PsyCap is therefore not analytically problematic in itself; however, its effectiveness cannot be assessed separately from the work environments in which it is applied. This central theoretical argument is revisited at several points throughout the paper.

### 2.3 Adaptation demands and organizational resources

Conservation of Resources (COR) theory proposes that people strive to obtain, retain, protect and foster the things they value, and that stress arises when resources are threatened with loss, are actually lost, or are not regained after significant investment (Hobfoll, 1989; Halbesleben et al., 2014). In its formalized organizational version, the theory rests on four principles: the primacy of resource loss, according to which loss is disproportionately more salient than gain; resource investment, according to which people must invest resources to protect against loss, recover from it and gain resources; the gain paradox, according to which resource gain becomes more salient in the context of loss; and desperation, according to which people whose resources are overstretched or exhausted enter a defensive mode (Hobfoll et al., 2018). These principles are accompanied by corollaries stating that people with greater resources are less vulnerable to resource loss and more capable of gain, whereas those with fewer resources are more vulnerable, and that initial loss begets further loss (Hobfoll, 2001; Hobfoll et al., 2018).

Two further COR concepts are central to this study. Resources do not exist in isolation but travel in resource caravans, and these caravans are fostered or impeded by resource caravan passageways: the environmental, organizational and social conditions that support, enrich and protect resources or, conversely, restrict and undermine them (Hobfoll, 2011). Structural disruption can threaten several resources simultaneously, including the relevance of skills, predictability, autonomy, continued employment and access to social and institutional support. Organizations and public institutions can therefore be read as passageways through which adaptation resources either reach workers or fail to do so. Because testing loss and gain cycles raises unresolved methodological issues (Sonnentag and Meier, 2024), these dynamics are used here as an interpretive lens rather than as hypotheses to be tested.

Job Demands-Resources (JD-R) theory contributes two more specific propositions (Demerouti et al., 2001; Bakker and Demerouti, 2017; Bakker et al., 2023). According to the dual-process proposition, job demands drive a health-impairment process leading to strain, whereas job resources drive a motivational process leading to engagement. According to the buffering proposition, job resources attenuate the effect of job demands on strain. JD-R treats demands and resources as characteristics of work rather than of the person, while allowing personal resources such as self-efficacy and optimism to function in a way similar to job resources (Bakker and Demerouti, 2017). Because JD-R has been characterized as a heuristic framework rather than a specific explanatory theory (Schaufeli and Taris, 2014), it is used here as a lens rather than as a model to be tested. Applications to digital work show that technology can operate both as a demand, for example through technostress, and as a resource (Dragano and Lunau, 2020; Pansini et al., 2023; Scholze and Hecker, 2024; Chuang et al., 2025). Job insecurity, including insecurity attributed to AI and automation, has likewise been conceptualized as a demand whose sources lie in organizational and societal conditions as well as in individual perceptions (De Cuyper et al., 2008; Shoss, 2017; Jiang et al., 2021; Klug et al., 2024; Walczok and Bipp, 2025).

This study borrows three elements from these theories. From COR, it takes the concept of resource caravan passageways and the corollary of asymmetric vulnerability. From JD-R, it takes the buffering proposition and the definition of demands and resources as features of work design. Taken together, these elements imply that when adaptation demands originate in the structural characteristics of work and resources are provided or withheld by organizational and institutional conditions, the absence of buffering resources is a property of the work system that can be deliberately managed rather than a personal problem for individuals to overcome. The Resilience Responsibility Gap, introduced below, specifies where such buffering resources are missing and who is responsible for providing them.

### 2.4 From adaptation responsibility to the Resilience Responsibility Gap

The term Resilience Responsibility Gap is introduced here to describe a mismatch between the adaptation challenges created by structural disruption and the range of resources, protections, opportunities for participation, and organizational responsibilities available to help people and their companies continue functioning sustainably.

Three characteristics must be considered to keep the gap within clearly defined limits. First, the gap must be measured relationally. Rather than focusing on whether an individual feels stressed, the emphasis should be on the mismatch between demands and resources. Stress may indicate a problem, but it is neither necessary for a gap to exist nor sufficient to confirm one. Second, there must be a clearly identifiable demand. If technologies are described without creating pressure to adapt, they are not considered a source of the gap, regardless of how revolutionary they may be. Third, the gap is multifaceted. Resources may include easily and quickly accessible retraining, employer-funded learning, job modifications, a psychologically safe environment, autonomy in decision-making, employee input, transparent management of AI, open dialog between labor and management, a social security net, support during periods of transition, and equal access for all communities. A gap emerges when any of these resources is missing, with the absence of each resource creating a distinct set of issues.

The Resilience Responsibility Gap is a discriminating construct: the coding rules specify the conditions under which a gap is not coded. When demands are severe but the necessary enabling conditions are present (clearly specified, appropriately distributed, and readily available) the result is a resilient configuration. This provides a counterexample that clarifies the conditions under which the gap does and does not emerge. The concept therefore differs from a general critique of the neoliberalization of responsibility because the Gap establishes specific boundary conditions for diagnosis. Figure 1 presents the resulting conceptual framework. Structural disruption creates adaptation demands, which are met by a particular configuration of individual, organizational, and policy/system resources. When these resources are insufficient, a Resilience Responsibility Gap emerges. When the available resources adequately meet the demands, resilience capacity is produced.

**Figure 1:**
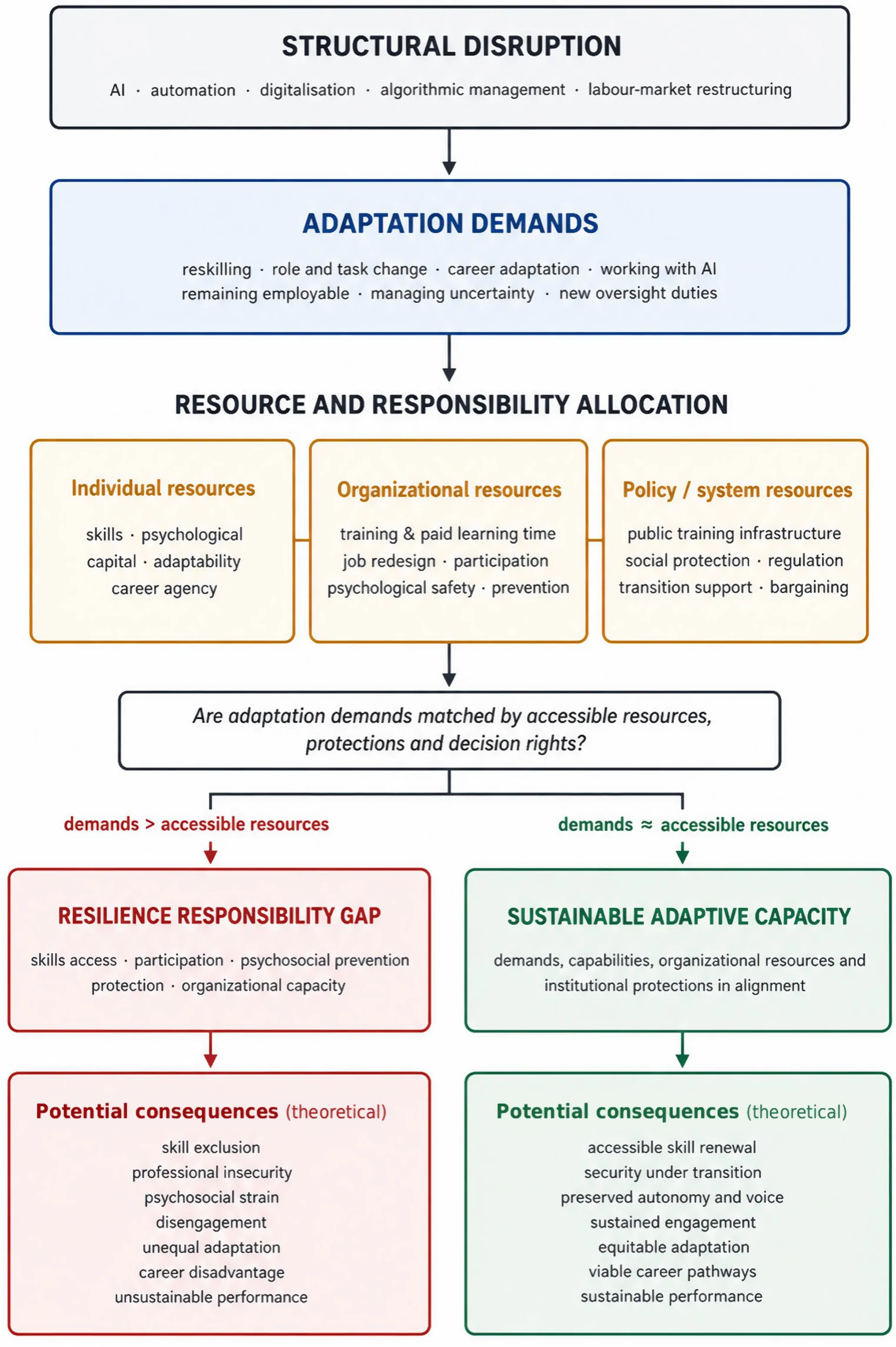
The resilience responsibility gap framework. The framework is conceptual. The relations between adaptation demands, resources and the gap were refined through the comparative document analysis, whereas the potential consequences shown in the figure are theoretical expectations derived from the literature and were not coded in the corpus. Arrows do not denote tested causal paths.

## 3 Methods

### 3.1 Research design

This study uses a structured, codebook-based comparative document analysis (Bowen, 2009) that combines qualitative content analysis (Hsieh and Shannon, 2005; Mayring, 2014; Schreier, 2014) with analytical sensitivity to policy problem representation and framing (Bacchi, 2012; Entman, 1993; van Hulst and Yanow, 2016). This research design is appropriate because the study does not focus on labor market outcomes, but on the policy problem of how responsibility for adaptation is allocated. Institutional documents function as a two-layered source. On the one hand, they describe changes in technology, working conditions, and participation in training, often drawing on evidence from numerous representative surveys. On the other hand, they provide legitimacy for particular policy arguments by specifying who should be involved in adaptation efforts, who should provide the necessary resources, how organizations should be encouraged to respond, and which risks should be managed by social institutions. Both layers were coded, and the analysis examined the contrast between how the documents described technological change and what they recommended regarding organizational adaptation. One example of this contrast is the recommendation that employees should receive continuous training alongside the acknowledgement that much of this training remains inaccessible to most workers. The study follows an analytical-conceptual design and does not aim to draw statistical conclusions or generalize from the sample to a wider population. Similarly, the corpus is neither representative of all institutional publications nor a random sample of institutional output. Frequency statistics were calculated and used solely as descriptive aids. They are not population statistics, nor is it suggested that they should be interpreted or used as such.

### 3.2 Corpus construction

The corpus was constructed purposively from the publications of international and European institutions whose mandate covers labor markets, employment, occupational safety and health, or digital transformation. A document was included if it (a) was produced by such an institution; (b) was published between 2021 and 2026, a period covering the recent diffusion of AI and algorithmic management in workplaces; and (c) was at least partly devoted to one or more of the following themes: AI and employment, digital transformation, changing skills demands, workforce adaptation, reskilling and upskilling, algorithmic management, occupational health, psychosocial risk, social dialog, worker participation, job quality, or structural change in the labor market. Three documents (D07, 2021; D08 and D09, 2022) predate the wide diffusion of generative AI and address digitalization and algorithmic management more broadly. They were retained because algorithmic management and digital work reorganization are part of the disruption examined here, but their findings are not read as evidence about generative AI specifically. Within these criteria, documents were chosen to cover six institutional perspectives: (1) labor market and employment policy; (2) skills, employability, reskilling and lifelong learning; (3) organizational change and digital transformation; (4) occupational health and psychosocial risk; (5) social dialog and worker participation; and (6) AI governance and algorithmic management. The aim was to maximize diversity of perspective and, in particular, to avoid restricting the analysis to the skills-policy genre, in which individualized framings are already common. The selection was deliberate rather than exhaustive, and the screening process (search terms and the number of candidate documents considered) was not systematically logged. The corpus should therefore be read as an analytically motivated purposive sample, not as the output of a reproducible systematic search.

The final corpus consists of 18 documents (Table 1): 9 issued by the OECD, 5 by Eurofound, 2 by EU-OSHA, 1 by the European Commission’s Joint Research Center in collaboration with EU-OSHA and Eurofound, and 1 by the International Labor Organization. By type, it includes 9 reports, 4 working papers (one of which, D18, is a literature review), 3 policy briefs, 1 policy review and 1 campaign guide. Fifteen documents form the core corpus; two are national country reviews used as comparative cases (D14, D15), and one provides a supplementary case on non-standard employment (D16). The predominance of OECD documents reflects the volume of OECD output devoted specifically to AI and the labor market in this period, including a dedicated working-paper series and country reviews, whereas the ILO is represented by a single briefing note. The corpus is therefore dominated by OECD and EU institutions (17 of 18 documents); developing and emerging economies enter only indirectly, through the ILO brief (D10) and comparative evidence reported in some OECD documents. Because these institutions share mandates that presuppose a legitimate role for public intervention in labor markets, the composition of the corpus is treated as a possible explanation of the findings in Section 5.

**Table 1:** Corpus characteristics (*N* = 18).

ID	Institution	Year	Document type	Primary focus	Structural disruption addressed
D01	OECD	2023	Report	Labor-market and employment policy	AI adoption across occupations; task and skill change
D02	OECD	2024	Working paper	Skill demand measurement	AI exposure reshaping skill demand
D03	OECD	2025	Policy brief	AI training supply and access	AI skill demand outpacing training supply
D04	OECD	2026	Policy brief	Skills policy synthesis	AI-driven change in skill requirements
D05	OECD	2026	Working paper	Skills systems and lifelong learning	AI transformation of skills and learning systems
D06	Eurofound	2024	Report	Organizational change and OSH	Advanced robotics/cobots restructuring tasks and control
D07	Eurofound	2021	Report	Organizational change and social dialog	Digitization (IoT, 3D printing, VR/AR) reorganizing work
D08	Eurofound	2022	Report	AI governance and algorithmic management	Digitization and algorithmic management as ethical restructuring
D09	JRC/EU-OSHA/Eurofound	2022	Working paper	Algorithmic management and psychosocial risk	Data-driven/algorithmic management of workers
D10	ILO	2026	Policy brief	Labor standards and social protection	AI transforming tasks, entry routes and job quality
D11	OECD	2026	Review	Comparative AI labor policy	AI diffusion requiring simultaneous policy responses
D12	EU-OSHA	2025	Report	Occupational health surveillance	Digital and climate change altering working conditions
D13	OECD	2025	Report	Organizational (SME) adoption capacity	AI adoption as organizational capability transition
D14	OECD	2025	Report	National labor market (Korea)	AI task/skill change amid aging and dualized labor market
D15	OECD	2025	Report	National labor market (Japan)	AI adoption under labor shortage and aging
D16	Eurofound	2024	Report	Non-standard work and social protection	Precarious and platform- mediated self-employment
D17	EU-OSHA	2023	Guideline	OSH prevention and worker participation	Digitalization, AI worker management, robotics, remote work
D18	Eurofound	2025	Working paper	AI, employment and social dialog	AI as socio-technical restructuring of work and voice

### 3.3 Coding framework

Coding was conducted using an *a priori* codebook developed before formal coding. For each field, the codebook included a clear operational definition, well-defined inclusion criteria, precise exclusion criteria, coding instructions, and a definitive list of permitted values. The individual document was selected as the unit of analysis, with coding covering any segment, section, table, or recommendation related to structural disruption and modification. The complete codebook, the coded matrix and a blank coding instrument are provided as Supplementary files S1, S2.

The twenty-eight fields were organized into five blocks. Bibliographic variables recorded the document, institution, publication year, title, and document type. Framing variables captured the scope of the structural disruption discussed, how the disruption was portrayed through its prevailing framing, and the primary stakeholder considered responsible for adapting to it. Responsibility variables separately recorded, in prose, the duties assigned to individual employees, organizations and employers, and policy and system actors. The intensity of these duties was assessed on the following scale: 0 = not included; 1 = referred to/trivial importance; 2 = of importance/moderate importance; and 3 = fundamental/of greatest importance. Resource and Risk variables recorded reskilling and upskilling, including who was expected to fund or provide these activities; psychological resources; explicitly discussed psychological capital; psychological risks; employee expression and participation (0 = absent; 1 = receives information; 2 = consulted; 3 = shares in decision making, negotiation or collective bargaining); equity and inclusion; adaptation resources; and adaptation demands. Synthesis variables documented the overall balance of responsibility; the Resilience Responsibility Gap score (0 = no significant disparity; 1 = slight/potential disparity; 2 = notable disparity); the reasoning behind the gap; a key piece of evidence or paraphrased excerpt supporting the argument; the page number or section reference where the excerpt appeared; and an analytical comment. Following the codebook instruction to code the strongest level supported by the document, the worker voice variable records the highest level of worker involvement that a document documents or endorses; a high code therefore does not imply that this level is widespread in the practices the document reports.

Coding was implemented at three distinct levels. Individual responsibility, such as adaptation through a worker’s own efforts, included skills acquisition, continuous learning, flexibility, and career management. Organizational responsibility covered the expectations and duties placed on employers, managers, and organizations in supporting adaptation, such as providing training, redesigning jobs to accommodate change, creating a psychologically safe environment for adaptation, ensuring transparency in the use of AI, consulting workers, providing adequate work, and supporting health and wellbeing. Policy/system responsibility referred to the duties of governments, policymakers, education systems, welfare providers, and labor market institutions in managing workforce transitions. These duties included providing social protection, regulation, public training infrastructure, rights protection, transition funding, collective bargaining arrangements, and active labor policies.

Two specific coding conventions were followed carefully to preserve analytical objectivity. First, the no-inference rule prohibited inferring workers’ psychological states, assigned responsibilities, or inherent resilience from vague references to technology. Only explicitly stated sentiments were recorded. Second, under the gap rule, a Resilience Responsibility Gap was coded only when a document clearly demonstrated a mismatch between demands and resources or between demands and responsibility. A gap was not coded merely because the document referred to disruption, stress, or changing skills demands.

Psychological content was identified with the same procedure and documentation requirements as responsibility content, using three fields. Psychological_Resources recorded psychological capacities or states that a document explicitly discussed as supporting continued functioning under disruption (e.g., resilience, hope, efficacy, optimism, adaptability, confidence, coping, psychological safety, trust), excluding technical skills without a psychological component. Psychosocial_Risks recorded explicitly identified adverse psychosocial consequences of structural change (e.g., technostress, burnout, anxiety, insecurity, surveillance, work intensification, loss of autonomy). PsyCap_Explicit was coded “Yes” only when the term psychological capital was used or when hope, efficacy, resilience and optimism were jointly framed as a combined construct; the mention of one or two components in isolation was coded “No.” As for all fields, each coded value was linked to a quotation or paraphrase with a page or section reference (Supplementary file S1, worksheet S5 Field-Level Evidence). Because this threshold requires explicit joint conceptualization, and the no-inference rule prohibits assembling the construct from dispersed components, the absence of PsyCap reported in Section 4.5 is partly a consequence of the coding definition. It should therefore be read as the absence of PsyCap as an explicit construct, not as the absence of its components, which are reported separately.

### 3.4 Analytical procedure

The analysis was conducted in two related phases. Phase 1 involved within-document coding, beginning with a summary assessment of the disruptive effects described in each document and the narrative used to explain that disruption. For each document, the executive summary, introduction and conclusion were read first to identify the dominant disruption and its framing; the sections addressing skills and training, workers, employers, policy, health and wellbeing, job quality, social dialog, participation and inequality were then read or searched. Depending on document length and structure, these sections were read in full or located through the table of contents and searches for the topic terms listed above. The three levels of responsibility were coded independently before the overall balance of responsibility within each document was assessed. This ensured that the summary judgment was informed by prior level-specific findings rather than allowing an initial summary judgment to shape the coding of individual levels. Adaptation demands and adaptation resources were recorded in separate fields and compared before the gap score was assigned.

In Phase 2, relevant content from the individual documents was consolidated into a matrix. Comparisons were then made across documents in relation to who was expected to adapt, where adaptation resources were located, whether responsibility was individualized or distributed, the balance between adaptation demands and resources, whether workers had a voice, the level of psychosocial risk, equity in adaptation, and the institutions in which adaptation practices were identified. Although the broader themes of responsibility and the adaptation gap were established before the analysis, the specific types of gap presented in Section 4.4 emerged inductively through comparisons across documents. A gap type was retained only when at least two documents contained overlapping page-level statements concerning that gap.

Analytical memos were written throughout both phases to document ambiguities, contradictions within individual documents, and emerging patterns. These memos helped identify the contradiction that became central to the findings: documents recommended continuous adaptation while simultaneously reporting that the conditions necessary to support it remained unmet.

### 3.5 Analytical rigor

Rigor was established primarily through procedural transparency (Lincoln and Guba, 1986; Morse et al., 2002; Tracy, 2010), using six strategies. First, an *a priori* codebook with operational definitions and inclusion and exclusion criteria was used. Second, a page-level audit trail linked every consequential coding decision to a quotation or paraphrase and a page or section reference. Third, within-document coding was kept separate from cross-document comparison, so that corpus-level patterns did not shape the coding of individual documents. Fourth, negative and counter-case analysis was used: documents whose demand–resource configurations did not show a gap were coded and analyzed rather than dismissed, and the gap rule was formulated to ensure this. Fifth, analytical memos documented uncertainty and alternative interpretations as they emerged. Sixth, borderline coding decisions were reviewed, and both the changes and their rationale were recorded.

Coding consistency was examined through two separate passes over the entire corpus, both conducted by the author at different points in time. Because the study is single-authored, this constitutes an intra-coder consistency check rather than an assessment of inter-coder reliability, and it cannot rule out stable interpretive tendencies of a single coder. The two passes differed in the response format of the two synthesis variables, not in their operational definitions. In the first pass, the Resilience Responsibility Gap was recorded as a binary flag (clear gap vs. no clear gap) and the responsibility balance as a compound label (e.g., “systemic/shared, policy-led”). In the second pass, the final response options described in Section 3.3 were applied. Consistency was therefore assessed on the categories the two formats share.

For the gap variable, second-pass scores were collapsed to the first-pass distinction (2 = clear gap; 0–1 = no clear gap). The passes agreed on 17 of 18 documents (94.4%; Cohen’s *κ* = 0.88; Krippendorff’s *α* = 0.88). The single divergence concerned D18, which was not flagged as a clear gap in the first pass but was scored 2 in the second, on the basis of the review’s own evidence of training and participation deficits (D18, pp. 32, 39, 59, 66). Within the second pass, D02 was additionally revised from 1 to 0 because the paper articulates no adaptation demand and therefore cannot establish a mismatch under the gap rule.

For the responsibility balance, the first-pass label matched the second-pass category in 10 of 18 documents (55.6%). All eight divergences involved adjacent non-individualized categories (e.g., shared vs. systemic), and no document was classified as individual-dominant in either pass (18/18). Divergences were resolved by rereading the relevant sections and recording the decision and its textual basis.

The pass comparison covered only these two synthesis variables. The responsibility intensity and worker voice variables were coded once, so no agreement statistic is available for them. To make these codes auditable, every coded value is linked to a quotation or paraphrase with a page or section reference (Supplementary file S1,worksheet S5 Field-Level Evidence). All agreement figures are reported as descriptive indicators of consistency rather than as evidence of inter-coder reliability. Findings that rest on these variables are therefore reported as descriptive results from a single coding pass, and the same qualification applies to the exact responsibility-balance category, on which the two passes agreed in 10 of 18 documents.

The author approached the corpus from a background in education research and comparative education policy, and with a prior expectation, informed by critical scholarship on resilience and employability, that institutional documents would tend to individualize responsibility for adaptation. To limit the influence of this expectation, the coding guide included an explicit caution against forcing the gap construct and required counter-cases to be coded and reported. The finding that no document allocated responsibility primarily to individual workers runs counter to this initial expectation.

## 4 Results

Six themes are reported. Frequencies are descriptive aids; the interpretive weight rests on the page-level evidence summarized for each theme. Table 2 presents the cross-document allocation of adaptation responsibility.

**Table 2:** Cross-document allocation of adaptation responsibility.

ID	Framing	Primary actor	Ind.	Org.	Pol.	Responsibility balance	Worker voice	Equity dimensions coded	Gap
D01	Mixed	Policy	1	2	3	Shared	3 Co-design	Skill; age; firm size	1
D02	Mixed	Policy	0	0	1	Unclear	0 None	Occupation; skill level	0
D03	Mixed	Policy	1	1	3	Systemic	0 None	Skill level; automation risk	2
D04	Mixed	Shared	1	2	3	Shared	2 Consultation	Firm size; skill level	1
D05	Mixed	Policy	2	2	3	Systemic	3 Co-design	Firm size; skill; gender; age	1
D06	Mixed	Shared	1	3	3	Shared	3 Co-design	Firm size; occupation; skill	2
D07	Mixed	Shared	1	3	3	Shared	3 Co-design	Qualification; age; contract	2
D08	Mixed	Policy	1	2	3	Systemic	3 Co-design	Gender; ethnicity; marginalized groups	1
D09	Mixed	Organization	1	3	2	Organizational	2 Consultation	Firm size; sector; contract	2
D10	Mixed	Policy	1	1	3	Systemic	3 Co-design	Country income; gender; age	0
D11	Mixed	Policy	1	2	3	Systemic	3 Co-design	Firm size; gender; disability	1
D12	Mixed	Organization	0	2	0	Organizational	2 Consultation	Occupation; firm size; contract	2
D13	Opportunity	Policy	1	2	3	Systemic	1 Information	Firm size; region; sector	1
D14	Mixed	Policy	1	2	3	Systemic	3 Co-design	Skill; age; gender; firm size	1
D15	Mixed	Shared	2	3	3	Shared	2 Consultation	Age; contract status; firm size	1
D16	Mixed	Policy	1	1	3	Systemic	3 Co-design	Contract status; gender; age	2
D17	Mixed	Shared	1	3	3	Shared	3 Co-design	Gender; migrant; disability	0
D18	Mixed	Shared	1	3	3	Shared	3 Co-design	Skill level; gender; contract	2

Ind./Org./Pol. = coded intensity of individual, organizational and policy/system responsibility (0 = absent, 1 = minor, 2 = moderate, 3 = central). Gap = Resilience Responsibility Gap (0 = no evident mismatch, 1 = possible/limited, 2 = clear). Framing = dominant framing of disruption. Primary actor = actor primarily expected to adapt. Worker voice = strongest level of involvement documented or endorsed in the document. The responsibility intensity and worker voice codes derive from a single coding pass; the exact responsibility-balance category showed 55.6% intra-coder agreement across the two passes (Section 3.5).

### 4.1 Theme 1: structural disruption creates a persistent adaptation imperative

The documents in the dataset generally portray AI and digitalization not as sudden developments, but as dynamic shifts affecting labor, skills, organizational structures, and labor relations. Of the 18 documents analyzed, 17 were categorized as mixed because they acknowledged the potential for both improvements and deterioration in job quality. D13 was the only exception, framing digitalization primarily as an opportunity to overcome the barriers faced by small and medium-sized enterprises when adopting new technologies. None of the documents presented the transition as unavoidable in a way that would diminish the importance of managing it. Instead, outcomes were depicted as dependent on specific implementation decisions. D08 emphasizes that the effects of technologies are not predetermined, but depend on how they are designed, introduced, and governed. D18 makes the same point in relation to job design, arguing that whether AI results in deskilling or empowerment “depends on how organizations redesign jobs, structure internal career ladders and support human-AI collaboration” (D18, pp. 68-69).

This contingent framing has a direct implication for the need to adapt. Because the effects of technology are presented as undetermined, the documents identify choices as the factors that shape those effects, and responsibility can be assigned for those choices. The corpus therefore establishes a continuing need for adaptation while also identifying who is expected to undertake it. This makes it possible to examine responsibility at a later stage.

These demands are recurrent rather than one-off. Machines shape the rhythm and content of work (D09), while the workforce must continually retrain and acquire new tools rather than simply completing a single transition from the age of manual labor (D05, D07). Management must continuously oversee systems in which humans and AI operate together while maintaining collaborative relationships with workers and machines. At the same time, redesigning production around robotics and cobotics increases cognitive and time-related demands (D06). D07 explicitly notes that work intensity was highest during the early stages of robotization and became more pronounced in workplaces where employers failed to provide adequate training policies or involve workers in pilot studies (D07, pp. 58, 66). However, it is already clear that the demand and resource dimensions are closely connected in practice.

Psychological outcomes are also acknowledged explicitly and consistently. Thirteen of the eighteen documents refer to psychological or related harms associated with structural change, including surveillance and monitoring, intensified workloads or time pressures, reduced self-determination or decision-making power, instability, fear of job loss, and information overload. As discussed in Section 4.4, this pattern provides important support for the analysis: the documents do not systematically suggest that transformation carries no psychological burden; rather, they show that protection from its negative effects is not universally available.

### 4.2 Theme 2: adaptation responsibility is distributed, but unevenly

The main finding was that none of the documents framed responsibility primarily in terms of individual choices and actions. Across the entire sample, the intensity variable “individual responsibility” was never coded as “central.” It was coded as absent in 2 documents, minor in 14, and moderate in 2 documents (D05, D15). In contrast, “policy/system responsibility” was classified as central in 15 documents, while “organizational responsibility” was classified as central in 6 and moderate in 8. Regarding the actor each document identified as primarily responsible for change, governments or policymakers were the most frequent, appearing in 10 documents. This was followed by shared responsibility in 6 documents and organizations or employers in 2. None of the documents identified workers as the group primarily responsible for change. The responsibility intensity and worker voice variables were coded in a single pass (Section 3.5), so the intensity distributions reported here are descriptive.

Regarding the overall balance of responsibility, 8 documents were classified as systemic, 7 as shared, 2 as organizational, and 1 as unclear. None were classified as individual. Both coding passes classified all 18 documents as non-individualized. Because the two passes agreed on the exact category in 10 of 18 documents (Section 3.5), this four-category distribution is reported as a descriptive result and should be interpreted cautiously; the classification of all 18 documents as non-individual-dominant held in both passes.

Where duties were listed for several actors without enforceable mechanisms or funding, higher intensity levels were not assigned. Possible explanations and limits of this finding, including the institutional mandates of the bodies represented in the corpus, are examined in Section 5.1.

Four possible ways in which work-related adaptation was framed were identified across the corpus. System-supported adaptation appeared when policy was recognized as the primary facilitator. This framing included references to social security, the activation of job creation, various regulations, and public education initiatives, as seen in D10, D11, and D16. Organization-led adaptation instead emphasized employer-led practices, including risk assessments, work redesign, accident prevention, and collaborative forums, as demonstrated in D09 and D12. Shared adaptation directly connected worker-level learning with support and provision from employers and institutions. This generally involved multilevel solutions, as seen in D01, D04, D15, D17, and D18. Finally, individualized adaptation places primary responsibility for managing adjustment on the individual worker. No document framed adaptation primarily at the individual level, although this framing was explicitly searched for.

### 4.3 Theme 3: reskilling dominates the institutional imagination of resilience

Regarding the adaptation of work and skills, skill renewal appeared in 17 of the 18 documents. It was presented as a shared responsibility in 11 documents, primarily as a policy- or system-level responsibility in 4, and as an employer responsibility in 2. In D12, a worker survey on occupational health and safety, training was presented as a welfare measure intended to support worker well-being and address stress. Consequently, this document did not frame reskilling or upskilling as a form of adaptation.

Across the corpus, skills were the most concretely specified adaptation resource. Training was more often linked to explicit targets, funding arrangements or participation measures, whereas job control, decision-making autonomy, employment stability and transparent algorithmic governance were more often addressed through general recommendations. A small number of documents questioned the separation of training from work design. D06 recommends that training “should form an integral part of sound change management and not be treated as a separate solution” (D06, p. 26), yet reports that in the cases examined training was “typically narrow in focus and it was not integrated into comprehensive change management” (D06, pp. 30, 47). D18 similarly places skills within organizational transformation rather than treating them as a stand-alone transition tool.

The documents also show that access to training is uneven. D01 reports that low-skilled workers face the highest risk of automation yet participate less in training than other workers, with an eight-percentage-point gap in the EU27 (D01, pp. 139, 164–166). D03 shows that one in three vacancies analyzed has high AI exposure, whereas only 0.3–5.5% of the training courses analyzed deliver AI content (D03, p. 3). Most existing provision targets AI professionals with above-average prerequisites, so the workers most vulnerable to AI-driven job loss are the least able to access it (D03, p. 4). D18 reports that only 15% of workers had received AI-related training, while four in ten reported a need to develop AI skills (D18, p. 32). D16 shows that economically dependent solo self-employed workers, who report the highest job strain and the least autonomy, have the lowest access to on-the-job training (24%, compared with 49% of employees; D16, pp. 24–26, Table 8). The evidence therefore points to an uneven distribution of access to the skills that are expected. When training opportunities become available, those who are already advantaged appear to be the first to benefit from them. This lies at the heart of Theme 4.

### 4.4 Theme 4: the resilience responsibility gap emerges where demands exceed enabling resources

Seven of the 18 documents contained a clear Resilience Responsibility Gap (D03, D06, D07, D09, D12, D16, D18). Eight documents contained a possible or limited gap (D01, D04, D05, D08, D11, D13, D14, D15), while three showed no obvious gap (D02, D10, D17). The Resilience Responsibility Gap was therefore prevalent but not inherent in the documents reviewed. It also followed a nonrandom distribution pattern, appearing most frequently in documents that provided clear empirical analyses of technology use in the workplace (D06, D07, D09, D12, D16). Because a gap could be coded only where a document reported evidence on implementation, this concentration partly reflects the coding design (see Section 5.2). The Gap was also identified in policy documents reporting their own data (D03) and in documents synthesizing literature on empirical findings concerning the effective implementation of the initiative (D18).

This pattern is particularly noteworthy. Documents describing what should be done generally paired adaptation demands with corresponding resources. Documents reporting establishment surveys, case studies, worker surveys or training-participation data, by contrast, showed where these pairings broke down in practice. The gap therefore emerges where ideal scenarios, shaped by a discourse of provision or demand, encounter evidence about how those scenarios are implemented in practice. Five subgroups were developed through inductive analysis, with each subtype supported by at least two documents. These subtypes are described below in inductive and descriptive terms rather than presented as a new formalized model.

- Skills Access Gap: High Learning Expectations, Unequally Accessible Provision. A policy objective should clearly not leave most workers without support. Nevertheless, this outcome emerges in D03. The brief provides evidence of an insufficient and narrowly focused supply of AI skills. AI training courses often target workers who are already qualified and provide inadequate financial incentives. Most courses do not focus sufficiently on AI-related skills to be meaningful. As a result, workers who may be vulnerable to AI-related redundancy are unlikely, for various reasons, to participate in the adaptation programs recommended to them. D18 presents a similar pattern: four in ten respondents report a need to develop AI skills, yet only 15% have received AI-related training (D18, p. 32). In D16, this problem affects dependent self-employed workers with particular severity. This pattern suggests a problem of reach rather than a lack of stated intent.
- Participation Gap: Major Technological Change, Limited Worker Voice. According to D06, the introduction of technology was primarily a management-led initiative rather than a process involving workers. Employees were informed rather than meaningfully consulted, even though the report called for their participation in implementation. In D07, employee involvement was limited to piloting technologies. The report emphasized the weakness of workplace social dialog, which generally went no further than meeting legal requirements to inform and consult employees. D09 found that only 24.1% of establishments using data-driven management technologies had discussed their possible impacts with the workforce (D09, p. 43). D18 reports that 60% of workers consider it unlikely that their employer would take employees’ views into account when introducing AI (D18, p. 39). Employee participation remained far behind direct consultation, unions often lacked the technical knowledge needed to contribute effectively, and only 20% of the unions surveyed had an AI-related collective agreement (D18, pp. 59, 66). AI governance was almost entirely absent from contracts. The issue is not a lack of financial or human resources; it is a question of power; whose views matter when changes are introduced? D06, D07 and D18 were coded at the highest worker-voice level because they document or endorse co-design and negotiation in some cases, whereas D09 was coded at the consultation level (Table 2). The participation gap lies in the distance between the involvement documented or endorsed and the involvement reported as typical.
- Psychosocial Prevention Gap: Growing Digital Demands, Diminishing Preventive Response. D09 provides the strongest evidence. The presence of technologies enabling data-driven management is associated with a higher prevalence of reported psychosocial risks, in particular time pressure and long or irregular working hours (D09, p. 41, Table 1), yet establishments using these technologies are less likely to have introduced measures to prevent psychosocial risks (D09, pp. 42-43, Table 2). At the same time, it is also “much less common” for these establishments to allow workers to organize their own activities (D09, pp. 41–43). D12 demonstrates these effects at the population level: 48% of the employees surveyed reported that digital tools regulated the pace of their work, and 44% experienced intense time pressure. However, only 45% said they had been consulted about stressful aspects of work, while 40% had access to professional support (D12, pp. 24, 30, 37-38). According to D06, company risk assessments were conducted only once and focused primarily on physical hazards. Psychosocial risk factors therefore frequently remained unnoticed in practice (D06, p. 26). The gap lies between the demands created by technological transformation and the preventive measures used to address them.
- Protection Gap: Structural Employment Risk, Weak Social Protection. D16 provides a clear example. Economically dependent solo self-employed workers experienced the highest workloads, the least job autonomy, the poorest expectations for the future, and the weakest social security provision. D14 reports a similar pattern in Korea, where non-regular workers have limited social protection (D14, p. 53). In 19 Member States (D16, p. 31), people without full employment lacked rights in at least one area of social security. Support measures introduced during the global pandemic were also delayed, provided in smaller amounts, and subject to more restrictive access requirements than those available to formally employed workers. The analysis concludes with an unusually direct assessment: formal inclusion does not guarantee access to social protection or provide sufficiently useful levels of support, particularly because many self-employed workers have limited financial resources, low earnings, and long gaps between payments. This reflects a disconnect between the risks encountered in the labor market and the social structures intended to compensate for them.
- Gap in Organizational Capacity. An organization cannot successfully undergo transformation if it lacks the resources required to do so. The same principle applies to employees who lack the resources needed to manage risk. D13 describes the barriers faced by SMEs in adopting technology as a persistent lack of capacity, including insufficient financial, informational, and infrastructural resources, as well as inadequate knowledge and skills to achieve the necessary transformation. As D12 shows, micro-enterprises are less likely than large enterprises to be aware of well-being and information programs or training opportunities (42% vs. 66%). The size-related differences observed across the three other preventive activities indicate that employees with the same needs (and therefore the same demand for assistance) receive different levels of managerial provision depending on the size of their enterprise (D12, p. 40). D06 and D09 provide similar evidence that technology is generally more accessible in larger firms. Across these documents, firm size is associated with differences in the provision of adaptation resources to workers.

Three documents were identified as showing no gap. Their structures provide some insight into what alignment looks like. The EU-OSHA guidelines document, D17, connects adaptation demands with the mechanisms intended to address them: a legal duty of prevention; risk assessments covering both the risks associated with digital work and those affecting workers’ psychosocial well-being; the provision of information to workers; requirements for consultation; participation in the design and implementation of new tools; and a range of supporting resources at the EU level. In D17’s allocation of responsibility, organizational and policy responsibilities were both central, while workers were included at the co-design stage. The ILO briefing note D10 grounds the demands it identifies in labor standards, social protection, active employment policy, a public training system responsive to workforce needs, and the principle of social dialog. It therefore does not identify demands without connecting them to corresponding institutional provision. D02 offers a slightly different counterexample. As a technical measurement paper, it identifies no adaptation demand and therefore, under the gap rule (Section 3.3), cannot establish a mismatch between demands and resources. It is thus a counter-case in which responsibility is absent rather than mismatched.

Far from being incidental, these examples form the foundation of the counter-analysis. They suggest that the central problem is not AI-driven change itself, but the specific configurations of power surrounding it. Alignment exists when employers have duties, workers have rights, public services are available and accessible, and individuals are not disadvantaged because they are engaged in non-standard employment.

### 4.5 Theme 5: psychological resources without a psychological framework

A wide range of psychosocial issues appears across the corpus, but these issues are not clearly connected to broader psychological concepts. Fifteen sources consider psychological resources, while 13 discuss psychosocial risks. The topics identified include trust in employers’ use of AI and in algorithms; autonomy and discretion; confidence; self-efficacy in learning; adaptability; social support; wellbeing; security; agency; coping mechanisms; and, in a small number of sources, psychological safety. Only two documents (D06, D15) apply the term resilience to workers; elsewhere it refers to systems, economies, households or funding instruments.

Other parts of the corpus indicate where these two dimensions are already being combined, even if the terminology does not fully align. Trust in a company’s use of AI (D15), autonomy in decision-making under algorithmic management (D09), a degree of self-efficacy associated with receiving instruction (D03, D05), and the psychological freedom to participate in the introduction of AI within an organization (D17, D18) are all psychological resources that operate through organizational mechanisms.

### 4.6 Theme 6: access to resilience resources is unequal

All 18 documents in the corpus (18/18) identified distributional differences in exposure, vulnerability, or access. These patterns appeared consistently across documents and dimensions, including skill and education, age, firm size, occupation, employment status or contract type, gender, sector, region, migration status, and, in the case of the ILO document, national income level. Three findings are particularly significant.

First, access is inversely related to exposure. Those most exposed to AI-driven change are often the least able to access the resources that could support them. D01 reports that automation primarily affects low-skilled jobs, while most low-skilled workers do not participate in training (D01, p. 139). D12 (p. 40) shows that 66% of employees in large firms are covered by some form of well-being training initiative, compared with only 42% of employees in micro firms. It also provides no way to distinguish training efforts among different groups within micro firms, despite broadly comparable technological demands. Economically dependent self-employed workers similarly face intense demands while having little access to relevant training and weak protections (D16).

Second, firm size acts as a systematic sorting mechanism. In this study, it simultaneously influences technology adoption (D06, D09, D13), access to training (D04, D13), and preventive capacity (D12). Employer capacity therefore affects whether state policies are translated into resources that workers can actually access. Publicly funded training grants, for example, may be impractical for small-business owners because they lack sufficient staff to release employees to attend training.

Third, employment status can limit access to nearly every resource identified above. Workers in atypical, temporary, platform-based, or sole-trader employment often receive less training (D07, D16), have weaker protection coverage (D16), exercise less agency (D16, D18), and receive less preventive support (D12).

In short, the central implication of the study can be restated as follows: workers may have identical adaptation needs, yet their access to the resources required to meet those needs can differ entirely. This is not because workers possess different capacities to adapt, but because they face unequal mismatches between demands and resources.

## 5 Discussion

The findings answer the four research questions as follows. Regarding RQ1, disruption was framed as mixed in 17 of 18 documents, with outcomes presented as contingent on design and governance choices rather than as technologically determined. Regarding RQ2, responsibility was allocated primarily to policy and system actors and to organizations, and no document was individual-dominant. Regarding RQ3, skills and training were the most prominent and most concretely specified resources, whereas participation, psychosocial prevention and social protection were addressed less operationally. Regarding RQ4, mismatches emerged mainly where documents reported implementation evidence and took five partly independent forms. Read through the problem-representation perspective adopted in the Methods (Bacchi, 2012), the documents largely represent AI-driven change as a problem of governing implementation rather than as a problem of individual workers’ deficits; what this representation leaves less visible is whether the resources they prescribe actually reach those who need them. The following sections interpret these answers and address the study’s integrative aim.

### 5.1 Rethinking workforce resilience as distributed adaptive capacity

The analysis starts from a negative finding. Across 18 documents from OECD- and EU-centered institutional sources, responsibility for adaptation was assigned well beyond workers themselves, with much of it located at the policy and system level. This finding should not be read as evidence that labor market discourse in general has abandoned individualized framings. The strongest alternative explanation is institutional mandate: the OECD, the ILO, Eurofound and EU-OSHA are bodies whose remit presupposes a legitimate role for public intervention, social dialog and prevention, so a systemic allocation of responsibility is partly what their mandates would lead one to expect. Documents produced by employer associations, consultancies, firms or national governments may distribute responsibility differently. The finding is therefore best read as evidence about how responsibility is framed at this institutional level, where the picture is more complex than the individualization thesis suggests in its simplest form, rather than as a general refutation of that thesis.

Two further qualifications apply. First, low individual responsibility does not mean that workers receive strong support. D03 treats policy responsibility as central, yet reports that much of the relevant training remains inaccessible to those who need it most (D03, p. 4). Second, responsibility may be distributed widely in name while remaining narrow in practice. Vincent et al. (2024) develop the concept of relational responsibilization in the context of diversity management to describe how responsibility dispersed across actors can leave obligations unevenly met. Adapted to the present context, the concept captures documents that list duties for employers, workers and governments without specifying who is accountable or how those duties will be funded. The corpus thus does not depict a fully functioning system of adaptation. The same documents that assign central duties also report that only a minority of those who need training receive it, that implementation decisions are made without involving those who experience their consequences, that preventive measures are weakest where digital demands are highest, and that workers outside standard employment may lack meaningful protection. Responsibility has not been transferred to individuals, but the needs that remain unmet after policy and organizational provision have not been redistributed either.

Workforce resilience can therefore be reconceptualized as distributed adaptive capacity: the capacity of a work system to absorb and respond to continuing structural change through an alignment between adaptation demands and the individual, organizational, and institutional resources available to meet them. Three characteristics of workforce resilience follow from this definition. First, it is relational because it depends on the fit between demands and resources rather than on either one in isolation. Second, it is multilevel because resources available at one level influence the usefulness of resources available at another. Analyses of intellectual capital in school communities similarly locate capital in collective and organizational stocks rather than solely in individuals (Gençtürk-Erdem and Kavrayıcı, 2025). Funded job training, for example, is a resource only when employers provide workers with the opportunity to participate; otherwise, its value is limited (Hobfoll, 2011). Similarly, union bargaining rights are a resource when unions possess the expertise needed to exercise them effectively; otherwise, they remain an unrealized potential resource (D18). Third, workforce resilience is distributional because a system may appear resilient overall while systematically failing to meet its responsibilities toward particular groups, as suggested by the negative relationship between exposure and access identified in Theme 6.

This interpretation is consistent with multilevel approaches to workplace resilience (Hartmann et al., 2020; Kuntz et al., 2017) and with work-design research showing that the workplace consequences of automation depend on design choices rather than on the technology itself (Parker and Grote, 2022). It is therefore necessary to state what distributed adaptive capacity renames and what it adds. It renames two existing ideas: the fit between demands and resources, which JD-R places at the center of strain and motivation, and the dependence of individual resources on the conditions that foster or impede them, which COR describes as resource caravan passageways (Hobfoll, 2011). It adds three elements. First, multilevel resilience models locate employee resilience in individual, team and organizational conditions (Hartmann et al., 2020; Kuntz et al., 2017; Näswall et al., 2019), and COR describes passageways as environmental conditions, but neither specifies which actor is obligated to provide a missing resource. Distributed adaptive capacity treats the allocation of responsibility as part of the construct, so that a resource shortfall is analyzed together with the actor expected to remedy it. Second, it extends the analysis to the institutional level, where social protection, regulation, bargaining rights and public training determine which organizational resources are available at all. Third, it adds a distributional dimension: a work system may display adequate resources on average while systematically failing particular groups.

These additions generate predictions that existing models do not make explicit. (a) Non-substitutability: because gap subtypes are partly independent, resources of one type should not compensate for the absence of another; additional training, for instance, should not reduce the adverse effects of a participation or protection gap. (b) Stratification: resilience indicators aggregated at the organizational level should mask gaps concentrated among workers with high exposure and low access, so that differences in adaptation outcomes between groups should be larger than organizational averages suggest. (c) Conditional individual effects: the association between individual psychological resources and sustainable adaptation should be weaker where a gap exists, as specified in the moderation proposition in Section 5.3. These predictions are offered as propositions for future research, not as findings of the present analysis.

### 5.2 The resilience responsibility gap

The gap construct presented in Figure 1 demonstrates its value by distinguishing among different configurations. Seven documents showed a clear gap, eight showed a limited gap, and three showed no gap. These counter-cases are what make the construct analytically useful. If every document had revealed a gap, the concept would simply restate that AI-driven change creates substantial demands. Three characteristics of the observed gaps deserve particular theoretical attention.

First, gaps emerge at the point of implementation. They are concentrated in documents reporting establishment surveys, case studies, worker surveys, and data on training participation, while they are less apparent in documents concerned solely with policy design. One interpretation is that policy design creates coherent pairings of demands and resources, whereas implementation data reveal where these pairings break down, so that the gap reflects a failure of delivery and reach rather than of discourse. An alternative explanation must also be considered: a gap can be observed only where a document reports implementation data, so its concentration in empirical documents is partly a product of the coding design. Policy-design documents may contain comparable mismatches that remain invisible because they report no evidence on delivery. The present corpus does not allow these two explanations to be separated.

Second, gaps are multidimensional and partly independent. The five subtypes (skills access, participation, psychosocial prevention, protection, and organizational capacity) do not occur together consistently. D03 demonstrates a pronounced skills-access gap even though policy responsibility is coded as central. D09 and D12 reveal psychosocial prevention gaps in contexts where organizational responsibility is clearly specified. D16 demonstrates a protection gap that no amount of training provision could resolve, consistent with research on social protection in platform-based and non-standard work (Behrendt et al., 2019; Rani and Furrer, 2021; Aloisi, 2022). A one-dimensional understanding of resilience resources (most notably, the idea that the solution is simply “more training”) cannot address gaps of the second, fourth, and fifth types.

Third, gaps are stratified. Across every subtype, the shortfall disproportionately affects workers who already have fewer resources, including those who are lower-skilled, less qualified, older, engaged in non-standard employment, economically dependent and self-employed, or employed by micro and small firms. This reflects the COR prediction of asymmetric vulnerability at the level of institutional provision rather than individual coping (Hobfoll et al., 2018). It also corresponds with established findings that active labor-market and training interventions frequently fail to reach precisely these groups (Card et al., 2018; Vooren et al., 2019; Escudero, 2018; Orfao and Malo, 2023; van den Berg et al., 2023; Hansen et al., 2025).

The participation gap requires particular attention because it is not a resource shortfall in the conventional sense. Workers in D06 and D07 were not merely provided with insufficient training; they were excluded from the decisions that created the demands they were subsequently expected to absorb. From a JD-R perspective, participation is a job resource. From a COR perspective, decision latitude is a resource whose loss is itself a stressor. From a governance perspective, participation is a right. D07 reports that work intensification was greater where employees were neither involved in piloting nor adequately trained (D07, p. 58). This descriptive pattern is consistent with a buffering role of participation and training, although it cannot establish an interaction effect. The finding is consistent with evidence that worker voice institutions shape the outcomes of technology adoption (Belloc et al., 2023; Haipeter et al., 2024; Collins and Atkinson, 2023) and with arguments that algorithmic management must be regulated as a condition of acceptable implementation (Adams-Prassl et al., 2023).

### 5.3 Psychological capital under structurally unequal conditions

The complete absence of PsyCap as an explicit construct across the 18 documents is a finding about the relationship between two bodies of literature rather than a deficiency in either one. It would be a mistake to conclude that psychological resources are unimportant for adaptation under conditions of structural disruption. There is substantial evidence supporting PsyCap and related constructs (Avey et al., 2011; Luthans and Youssef-Morgan, 2017; Loghman et al., 2023; Rudolph et al., 2017), and the corpus repeatedly refers to their components without bringing them together within a single framework. These include confidence in participating in learning, trust in an employer’s use of AI, adaptability, agency, and social support. The constructs are present, but the framework is absent. The pattern points to a disconnect between two literatures. Institutional documents specify many preconditions of adaptation, such as access to training, participation, protection and prevention, but lack a framework for the psychological resources through which adaptation operates. Psychological research offers well-developed constructs such as PsyCap, and multilevel resilience research models organizational conditions (Hartmann et al., 2020; Kuntz et al., 2017), but it rarely extends to the institutional conditions that determine whether resources are available at all.

It would be equally mistaken to conclude that institutions should therefore “teach PsyCap.” Such an inference would reproduce precisely the tendency questioned by this analysis: turning a structural mismatch into an objective for worker development. The evidence suggests that psychological capacity is not the binding constraint when the gap concerns access to training, decision-making rights, preventive infrastructure, or social protection.

A more defensible position is that psychological capital is structurally conditioned. Its development, use, and benefits depend on circumstances beyond the individual’s control. Hope, understood as the combination of goal-directed agency and pathways thinking (Snyder, 2002), requires credible pathways; in career terms, this means visible and accessible transition opportunities, which active labor-market policies and internal career ladders may either provide or withhold. Self-efficacy develops primarily through mastery experiences (Bandura, 1997), which require training that is both accessible and feasible within workers’ time constraints, and perceived employability operates through job security rather than independently of it (De Cuyper et al., 2008). Studies of teachers show that occupational self-efficacy and resilience mediate the relationship between occupational stress and subjective well-being (Çiçek et al., 2026), and that professional resilience is positively related to positive and negatively related to negative work emotions (Yön and Dilekçi, 2026), suggesting that these resources operate within, rather than independently of, the demands of the work setting. Occupational stress and occupational resilience have likewise been found to predict teachers’ job performance (Şimşek and Dilekçi, 2025). From this perspective, the difference between the share of workers who report needing AI skills and the share who have received AI-related training (D18, p. 32) can be read as limiting opportunities for such experiences. Optimism requires a credible basis for expecting positive outcomes, which is difficult to maintain when 60% of workers consider it unlikely that their views on AI implementation will be taken into account (D18, p. 39). Individual resilience may likewise be depleted by chronic demands in the absence of recovery resources, as the psychosocial prevention gap identified in D09 and D12 suggests.

This leads to a testable proposition for future research: the relationship between individual psychological capital and sustainable adaptation outcomes is moderated by the configuration of organizational and institutional resources. This relationship should be stronger when demands and resources are aligned and weaker when a Resilience Responsibility Gap exists. Under gap conditions, PsyCap may still predict short-term coping but fail to predict sustainable adaptation because coping is being used as a substitute for missing structural resources. This is the mechanism anticipated by critical literature when resilience is described as compensatory (Joseph, 2013; Gill and Orgad, 2018; Suslovic and Lett, 2024), expressed in a form that occupational health psychology can test. It also has an important implication for measurement: studies examining workforce resilience under AI-driven change should assess the resource environment, including access to training, decision latitude, participation, employment security, and preventive provision, alongside individual capacities, rather than treating the environment merely as a covariate or leaving it as unmeasured context.

### 5.4 Alignment as the central organizational and policy problem

If resilience is understood as distributed adaptive capacity, the central question becomes one of alignment. A resilient workforce depends on a proper fit among adaptation demands, individual capabilities, organizational resources, and institutional protections. The Resilience Responsibility Gap describes a failure of this alignment. Evidence from the corpus shows that these failures are generally specific rather than universal: a training system that reaches the wrong people, an implementation process that excludes participation, a prevention framework that has not been expanded to address digital demands, a social protection boundary limited to standard employment, or an employer too small to release workers for training.

The prominence of skills in the corpus has a further implication. When the mismatch between a worker and a changed job is defined as a skills deficit, the solution becomes teaching the worker rather than redesigning the job, even when institutions accept responsibility for providing training. Other enabling conditions, such as job control, autonomy, employment stability, algorithmic governance, psychological safety, the financial capacity to engage in learning and participation, then receive less operational attention. The tension noted in Section 4.3, whereby documents that promote training also question its separation from work design, suggests that this emphasis is contested within institutional discourse itself.

This specificity has practical value. It suggests that the appropriate diagnostic question is not “are our workers resilient?” but “which resource is missing, who lacks access to it, and who controls it?” It also indicates that interventions must correspond to the particular type of gap. Funding training does not resolve a participation gap, while increasing the volume of lifelong learning without improving its reach reproduces existing inequalities (Kim and Park, 2020). Consultation does not address a protection gap. Similarly, regulating algorithmic management does not, on its own, resolve a capacity gap in small firms.

Finally, alignment changes how the temporal dimension is understood. Structural disruption creates an adaptation burden before any resilience outcome emerges. Analytically, this means that the relevant causal sequence begins earlier than most resilience research assumes: who creates the disruption, who is expected to adapt, and who controls the resources required for adaptation? Individual differences enter this sequence relatively late, so research designs that hold the earlier stages constant may be unable to show how far adaptation outcomes depend on them.

## 6 Practical and policy implications

The main practical contribution of the framework is diagnostic: it matches interventions to the type of gap rather than recommending more resources in general. Several of the recommendations below are already made in the corpus (e.g., D06, D09, D17); the added value of the Resilience Responsibility Gap lies in linking them to specific gap subtypes and to the actors responsible for closing them. Table 3 summarizes this correspondence.

**Table 3:** Gap subtypes, responsible actors and matching interventions.

Gap subtype	Missing resource	Primary responsible actors	Corpus evidence	Matching intervention	Does not close the gap on its own
Skills access	Accessible, time-feasible, AI-relevant training	Policy actors; employers	D03, D16, D18	Targeting of under-reached groups; paid learning time; low-prerequisite entry points	Increasing training volume
Participation	Worker voice in technology design and implementation	Employers; social partners	D06, D07, D09, D18	Consultation and co-design before adoption; technical capacity of worker representatives	Training provision
Psychosocial prevention	Risk assessment and prevention covering digital demands	Employers; OSH authorities	D06, D09, D12	Psychosocial risk assessment of digital tools; autonomy treated as a design parameter	Reskilling
Protection	Effective social protection for non-standard workers	Policy actors	D14, D16	Effective rather than formal access to protection; transition support	Training; consultation
Organizational capacity	Financial, informational and staffing capacity	Policy actors; intermediary bodies	D06, D09, D12, D13	Capacity support; cover for release time; shared services	Cost subsidies or regulation alone

Corpus evidence lists documents with page-referenced statements on each subtype; the right-hand column indicates interventions that, on the evidence of the corpus, do not close the gap on their own.

For organizations. Adaptation to AI should not be framed solely as an obligation for workers to reskill. Before introducing AI, organizations can conduct an adaptation-demand assessment to identify which roles will experience changes in tasks, work pace, autonomy, or performance evaluation and determine the resources each affected group will need. This assessment need not be a new instrument: it can extend the risk evaluation and worker consultation that EU occupational safety and health law already requires when new technologies are planned and introduced (European Union, 2008), by adding adaptation demands and the resources available to each affected group. Training must be both accessible and feasible within workers’ available time. Paid learning and time away from regular duties are as important as the availability of courses, particularly because the most frequently documented failure in the corpus is the provision of training without meaningful access to it. Employees should be involved in AI implementation during the design stage rather than merely informed after decisions have been made. Evidence from the corpus connects participation and training with lower levels of perceived work intensification during the early stages of adoption (D07) and identifies top-down implementation as a recurring source of mismatch (D06). Autonomy and psychological safety should be treated as design parameters because algorithmic management and monitoring systems can erode both by default. Psychosocial risks should also be assessed alongside technical risks, as the corpus shows that preventive measures lag precisely where digital demands are greatest (D09, D12). In school settings, equity-oriented leadership has been found to combine support for staff with a positive organizational climate while being constrained by financial and material barriers (Kavrayıcı, 2024), which illustrates that equitable provision depends on organizational capacity. Finally, organizations should monitor whether adaptation resources are distributed equitably across occupational groups, contract types, and skill levels rather than assuming that a company-wide average accurately represents the circumstances of individual workers.

For policy actors. Lifelong-learning infrastructure should be evaluated according to its reach rather than its volume. The evidence in D03 and D18 concerns who receives training, not how much training is available. Incentive structures that fail to target AI-relevant content or provide entry points for people with fewer prerequisite skills will reproduce existing inequalities. SMEs require support that addresses capacity rather than cost alone because firm size influences whether policy provision translates into resources that workers can actually access (D12, D13). Protection for precarious, platform-based, and self-employed workers must focus on effective rather than merely formal access, as the conclusion of D16 states directly. Transition support, active labor-market policies, and institutions for social dialog should all be treated as part of the infrastructure required for adaptation. The governance of algorithmic management belongs within the same policy framework as skills because decision-making rights and transparency are adaptation resources, not separate ethical concerns. Worker wellbeing should also be an explicit objective of digital transformation policy rather than a downstream occupational health issue, particularly because the strongest psychosocial evidence in the corpus comes from documents outside the skills-policy genre.

For workforce resilience research. The field’s dominant question, which employees are resilient, should be supplemented by questions that this analysis suggests must be addressed first: Which environments enable resilience? Which actors control adaptation resources? Who bears the financial and time-related costs of reskilling? How are structural risks distributed? Under what conditions does psychological capital lead to sustainable rather than compensatory adaptation? Methodologically, this approach favors multilevel and longitudinal research designs in which the resource environment is measured rather than assumed, as well as comparative designs that make use of variation across institutional configurations.

## 7 Limitations and future research

Ten limitations should be considered when interpreting these findings.

First, the corpus was constructed purposively rather than exhaustively. The 18 documents were selected for their analytical relevance and diversity of perspectives, not to represent the entire population of institutional publications. Different selection decisions could therefore produce different frequencies, and the descriptive counts should be interpreted with this in mind. In addition, the screening process was not systematically logged, and the corpus is dominated by OECD and EU institutions (17 of 18 documents), so the findings cannot be generalized to developing or emerging economies.

Second, policy-design documents describe intentions and recommendations rather than implementation. For these documents, a central allocation of responsibility to policy actors is a discursive allocation, not confirmation that resources have been provided. Documents that report implementation evidence allow some mismatches to be observed, but this evidence is itself selected and framed by the reporting institutions (see the third limitation).

Third, and relatedly, institutional discourse may differ from workers’ lived experiences. Although the corpus includes substantial survey evidence concerning worker experiences, this evidence is available only after being selected and framed by the institutions reporting it.

Fourth, coding necessarily involves interpretation. Assessments of dominant framing, the intensity of responsibility, and the presence of a gap required reading across lengthy documents and weighing different passages against one another. The codebook’s exclusion criteria, the no-inference rule, and the page-level audit trail limit the scope of this interpretation but cannot eliminate it entirely. Coding was conducted by a single author; consistency was checked across two passes only for the gap and responsibility-balance variables. Findings based on the responsibility intensity and worker voice variables are therefore descriptive results from a single coding pass, and the exact responsibility-balance category should be interpreted cautiously.

Fifth, the study does not measure individual psychological resilience or psychological capital. It examines how institutional discourse constructs the conditions for adaptation. Its claims about PsyCap concern the presence of the construct within that discourse rather than its distribution among workers or its effects on workforces.

Sixth, the absence of PsyCap from these documents does not mean that PsyCap is absent from actual workforce adaptation processes. Institutional genres use their own vocabularies, and this absence is best understood as a disciplinary disconnect rather than as evidence about workers themselves.

Seventh, international and European institutions are heavily overrepresented compared with national and sub-national policy actors, sectoral bodies, employer organizations, and firms. The finding that responsibility is not individualized may therefore be partly attributable to this particular institutional level. Testing this finding against employer-side and consultancy discourse is an evident next step.

Eighth, the conclusions are conceptual and analytical rather than causal. The Resilience Responsibility Gap framework was developed through a comparative analysis of documents. It is a conceptual framework requiring further testing, not a validated causal model. The moderation proposition presented in Section 5.3 is offered in this context.

Ninth, the gap finding is partly confounded with document type. Because mismatches can be observed only where implementation data are reported, the concentration of gaps in empirical documents may reflect the coding design as well as the substance of institutional provision.

Tenth, only English-language documents were analyzed, and three documents (D07, D08, D09) predate the wide diffusion of generative AI. National-language documents and more recent publications may frame responsibility differently.

Four directions for future research follow. (a) Construct validation: developing and validating establishment-level measures of the gap subtypes by combining indicators of demands with indicators of resources and decision-making rights. (b) Moderation testing: conducting multilevel studies to examine whether the relationship between PsyCap and adaptation becomes weaker under gap conditions. (c) Comparative institutional analysis: expanding the document corpus to include national, sectoral, and employer-side sources to determine whether the non-individualized pattern is specific to international institutions. (d) Longitudinal implementation research: following technology implementation from the initial decision through to its outcomes and measuring participation, access to training, and psychosocial provision as they occur rather than retrospectively.

## 8 Conclusion

Under conditions of AI-driven structural disruption, workforce resilience is not simply a quality that individuals either possess or lack. Instead, it emerges from the alignment between adaptation demands and the individual, organizational, and institutional resources available to address them. A Resilience Responsibility Gap arises when individuals and other actors are expected to adapt without adequate access to skills development, participation, psychological safety, employment protection, or institutional support.

The analysis indicates that, in this OECD- and EU-dominated corpus of international institutional documents, responsibility for adaptation is not individualized. None of the 18 documents identified workers as the primary bearers of adjustment, and policy-level responsibility was central in most. Nevertheless, clear mismatches between demands and resources appeared in seven documents, and partial mismatches in eight. These mismatches were concentrated in documents reporting what occurs in workplaces rather than what should occur. This pattern is consistent with a failure of institutional reach rather than of institutional intent, although it may also partly reflect the fact that mismatches can be observed only where implementation data are reported. In either case, the shortfall disproportionately affects workers who already have fewer resources.

The central organizational question should therefore move from “How can we make workers more resilient?” to “How should adaptation demands, resources, and responsibilities be distributed so that resilience is sustainable rather than compensatory?” This distinction is important because the two questions lead to different interventions. The first focuses on training the individual, while the second requires an examination of who creates the disruption, who is expected to absorb its effects, and who controls the resources needed to do so. Psychological capital continues to have an important role in the second formulation, but as a capacity whose development and benefits are structurally conditioned rather than as a substitute for the conditions themselves.

## Data Availability

The original contributions presented in the study are included in the article and its Supplementary material. Supplementary File S1 contains the corpus index, the codebook, the coding quick guide, the complete coded matrix, the comparison of the two coding passes and the field-level evidence with page references. Supplementary File S2 contains the blank coding instrument. All documents in the corpus are publicly available at the URLs listed in Appendix A. The study materials are also available on ScienceDB at https://www.scidb.cn/en/s/6biYnm. Further inquiries can be directed to the corresponding author.

## References

[ref1] Acemoglu D. Restrepo P. (2019). Automation and new tasks: how technology displaces and reinstates labor. J. Econ. Perspect. 33, 3–30. doi: 10.1257/jep.33.2.3

[ref2] Adams-Prassl J. Abraha H. Kelly-Lyth A. Silberman M. S. Rakshita S. (2023). Regulating algorithmic management: a blueprint. Europ. Labour Law J. 14, 124–151. doi: 10.1177/20319525231167299

[ref3] Aloisi A. (2022). Platform work in Europe: lessons learned, legal developments and challenges ahead. Europ. Labour Law J. 13, 4–29. doi: 10.1177/20319525211062557

[ref4] Avey J. B. Reichard R. J. Luthans F. Mhatre K. H. (2011). Meta-analysis of the impact of positive psychological capital on employee attitudes, behaviors, and performance. Hum. Resour. Dev. Q. 22, 127–152. doi: 10.1002/hrdq.20070

[ref5] Bacchi C. (2012). “Introducing the “what’s the problem represented to be?” approach,” in Engaging with Carol Bacchi: Strategic Interventions and Exchanges, eds. Bletsas A. Beasley C. (Adelaide: University of Adelaide Press), 21–24.

[ref6] Bakker A. B. Demerouti E. (2017). Job demands-resources theory: taking stock and looking forward. J. Occup. Health Psychol. 22, 273–285. doi: 10.1037/ocp0000056, 27732008

[ref7] Bakker A. B. Demerouti E. Sanz-Vergel A. I. (2023). Job demands-resources theory: ten years later. Annu. Rev. Organ. Psychol. Organ. Behav. 10, 25–53. doi: 10.1146/annurev-orgpsych-120920-053933

[ref8] Bandura A. (1997). Self-Efficacy: The Exercise of Control. New York, NY: W. H. Freeman.

[ref9] Behrendt C. Nguyen Q. A. Rani U. (2019). Social protection systems and the future of work: ensuring social security for digital platform workers. Int. Soc. Secur. Rev. 72, 17–41. doi: 10.1111/issr.12212

[ref10] Belloc F. Burdin G. Landini F. (2023). Advanced technologies and worker voice. Economica 90, 1–38. doi: 10.1111/ecca.12446

[ref11] Benlian A. Wiener M. Cram W. A. Krasnova H. Maedche A. Möhlmann M. . (2022). Algorithmic management: bright and dark sides, practical implications, and research opportunities. Bus. Inf. Syst. Eng. 64, 825–839. doi: 10.1007/s12599-022-00764-w

[ref12] Bowdler M. Lahti H. Jelenko M. Buresti G. Valtonen T. (2026). Algorithmic management and psychosocial risks at work: an emerging occupational safety and health challenge. Scand. J. Work Environ. Health 52, 1–5. doi: 10.5271/sjweh.4270, 41353777 PMC12766920

[ref13] Bowen G. A. (2009). Document analysis as a qualitative research method. Qual. Res. J. 9, 27–40. doi: 10.3316/QRJ0902027

[ref14] Brown W. (2015). Undoing the Demos: Neoliberalism’s Stealth Revolution. New York, NY: Zone Books.

[ref15] Card D. Kluve J. Weber A. (2018). What works? A meta analysis of recent active labor market program evaluations. J. Eur. Econ. Assoc. 16, 894–931. doi: 10.1093/jeea/jvx028

[ref16] Chuang Y.-T. Chiang H.-L. Lin A.-P. (2025). Insights from the job demands-resources model: AI’S dual impact on employees’ work and life well-being. Int. J. Inf. Manag. 83:102887. doi: 10.1016/j.ijinfomgt.2025.102887

[ref17] Çiçek İ. Sipahioğlu M. Dilekçi Ü. (2026). The mediating role of occupational self-efficacy and resilience in the relationship between occupational stress and subjective well-being in teachers: validation of the Turkish version of the teacher occupational self-efficacy-short form. Psychol. Sch. 63, 37–52. doi: 10.1002/pits.70073

[ref18] Collins P. Atkinson J. (2023). Worker voice and algorithmic management in post-Brexit Britain. Transfer Eur. Rev. Labour Res. 29, 37–52. doi: 10.1177/10242589221143068

[ref19] Dawkins S. Martin A. Scott J. Sanderson K. (2013). Building on the positives: a psychometric review and critical analysis of the construct of psychological capital. J. Occup. Organ. Psychol. 86, 348–370. doi: 10.1111/joop.12007

[ref20] De Cuyper N. Bernhard-Oettel C. Berntson E. De Witte H. Alarco B. (2008). Employability and employees’ well-being: mediation by job insecurity. Appl. Psychol. 57, 488–509. doi: 10.1111/j.1464-0597.2008.00332.x

[ref21] Demerouti E. Bakker A. B. Nachreiner F. Schaufeli W. B. (2001). The job demands-resources model of burnout. J. Appl. Psychol. 86, 499–512. doi: 10.1037/0021-9010.86.3.499, 11419809

[ref22] Dilekçi Ü. Limon İ. Katıtaş S. (2026). How shared instructional leadership is associated with teacher job satisfaction: the role of self-efficacy and professional resilience. BMC Psychol. 14:890. doi: 10.1186/s40359-026-04671-7, 42067942 PMC13276956

[ref23] Dobbins T. Plows A. (2023). Contesting the politics of neoliberal resilience: regional labour market resilience from a workers’ perspective. Reg. Stud. 57, 26–40. doi: 10.1080/00343404.2022.2052274

[ref24] Dragano N. Lunau T. (2020). Technostress at work and mental health: concepts and research results. Curr. Opin. Psychiatry 33, 407–413. doi: 10.1097/YCO.0000000000000613, 32324623

[ref25] Edmondson A. C. (1999). Psychological safety and learning behavior in work teams. Adm. Sci. Q. 44, 350–383. doi: 10.2307/2666999

[ref26] Edmondson A. C. Bransby D. P. (2023). Psychological safety comes of age: observed themes in an established literature. Annu. Rev. Organ. Psychol. Organ. Behav. 10, 55–78. doi: 10.1146/annurev-orgpsych-120920-055217

[ref27] Eloundou T. Manning S. Mishkin P. Rock D. (2024). GPTs are GPTs: labor market impact potential of LLMs. Science 384, 1306–1308. doi: 10.1126/science.adj0998, 38900883

[ref28] Entman R. M. (1993). Framing: toward clarification of a fractured paradigm. J. Commun. 43, 51–58. doi: 10.1111/j.1460-2466.1993.tb01304.x

[ref29] Epel E. S. Crosswell A. D. Mayer S. E. Prather A. A. Slavich G. M. Puterman E. . (2018). More than a feeling: a unified view of stress measurement for population science. Front. Neuroendocrinol. 49, 146–169. doi: 10.1016/j.yfrne.2018.03.001, 29551356 PMC6345505

[ref30] Escudero V. (2018). Are active labour market policies effective in activating and integrating low-skilled individuals? An international comparison. IZA J. Labor Policy 7:4. doi: 10.1186/s40173-018-0097-5

[ref31] European Union (2008). Council Directive 89/391/EEC of 12 June 1989 on the Introduction of Measures to Encourage Improvements in the Safety and Health of Workers at Work. Brussels: European Union.

[ref32] Fejes A. (2010). Discourses on employability: constituting the responsible citizen. Stud. Contin. Educ. 32, 89–102. doi: 10.1080/0158037X.2010.488353

[ref33] Felten E. Raj M. Seamans R. (2021). Occupational, industry, and geographic exposure to artificial intelligence: a novel dataset and its potential uses. Strateg. Manag. J. 42, 2195–2217. doi: 10.1002/smj.3286

[ref34] Ganster D. C. Rosen C. C. (2013). Work stress and employee health: a multidisciplinary review. J. Manag. 39, 1085–1122. doi: 10.1177/0149206313475815

[ref35] Gençtürk-Erdem E. Kavrayıcı C. (2025). Intellectual capital in school communities: a mixed-method study. KEDI J. Educ. Policy 22, 47–74. doi: 10.22804/KJEP.2025.22.1.003

[ref36] Gill R. Orgad S. (2018). The amazing bounce-backable woman: resilience and the psychological turn in neoliberalism. Sociol. Res. Online 23, 477–495. doi: 10.1177/1360780418769673

[ref37] Haipeter T. Wannöffel M. Daus J.-T. Schaffarczik S. (2024). Human-centered AI through employee participation. Front. Artif. Intellig. 7:1272102. doi: 10.3389/frai.2024.1272102, 38496830 PMC10941644

[ref38] Halbesleben J. R. B. Neveu J.-P. Paustian-Underdahl S. C. Westman M. (2014). Getting to the “COR”: understanding the role of resources in conservation of resources theory. J. Manag. 40, 1334–1364. doi: 10.1177/0149206314527130

[ref39] Hansen A. K. Christiansen L. Lassen A. H. (2025). Technology isn’t enough for industry 4.0: on SMEs and hindrances to digital transformation. Int. J. Prod. Res. 63, 6585–6605. doi: 10.1080/00207543.2024.2305800

[ref40] Hartmann S. Weiss M. Newman A. Hoegl M. (2020). Resilience in the workplace: a multilevel review and synthesis. Appl. Psychol. 69, 913–959. doi: 10.1111/apps.12191

[ref41] Hobfoll S. E. (1989). Conservation of resources: a new attempt at conceptualizing stress. Am. Psychol. 44, 513–524. doi: 10.1037/0003-066X.44.3.513 2648906

[ref42] Hobfoll S. E. (2001). The influence of culture, community, and the nested-self in the stress process: advancing conservation of resources theory. Appl. Psychol. 50, 337–421. doi: 10.1111/1464-0597.00062

[ref43] Hobfoll S. E. (2011). Conservation of resource caravans and engaged settings. J. Occup. Organ. Psychol. 84, 116–122. doi: 10.1111/j.2044-8325.2010.02016.x

[ref44] Hobfoll S. E. Halbesleben J. Neveu J.-P. Westman M. (2018). Conservation of resources in the organizational context: the reality of resources and their consequences. Annu. Rev. Organ. Psychol. Organ. Behav. 5, 103–128. doi: 10.1146/annurev-orgpsych-032117-104640

[ref45] Hsieh H.-F. Shannon S. E. (2005). Three approaches to qualitative content analysis. Qual. Health Res. 15, 1277–1288. doi: 10.1177/1049732305276687, 16204405

[ref46] Hui X. Reshef O. Zhou L. (2024). The short-term effects of generative artificial intelligence on employment: evidence from an online labor market. Organ. Sci. 35, 1977–1989. doi: 10.1287/orsc.2023.18441, 19642375

[ref47] Jarrahi M. H. Newlands G. Lee M. K. Wolf C. T. Kinder E. Sutherland W. (2021). Algorithmic management in a work context. Big Data Soc. 8:20539517211020332. doi: 10.1177/20539517211020332

[ref48] Jiang L. Xu X. Wang H.-J. (2021). A resources-demands approach to sources of job insecurity: a multilevel meta-analytic investigation. J. Occup. Health Psychol. 26, 108–126. doi: 10.1037/ocp0000267, 33119333

[ref49] Joseph J. (2013). Resilience as embedded neoliberalism: a governmentality approach. Resilience 1, 38–52. doi: 10.1080/21693293.2013.765741

[ref50] Kavrayıcı C. (2024). Social justice leadership in school settings: a qualitative study. Res. Educ. Administ. Leader. 9, 232–266. doi: 10.30828/real.1431311

[ref51] Kavrayıcı C. Shikhkamalova V. Tamdoğan Evliyaoğlu A. (2024). Psychological capital as a predictor of professional resilience capacities of teachers in public schools. CADMO 2, 71–77. doi: 10.3280/cad2024-002005

[ref52] Kellogg K. C. Valentine M. A. Christin A. (2020). Algorithms at work: the new contested terrain of control. Acad. Manag. Ann. 14, 366–410. doi: 10.5465/annals.2018.0174

[ref53] Kim J. Park C.-Y. (2020). Education, skill training, and lifelong learning in the era of technological revolution: a review. Asian Pac. Econ. Lit. 34, 3–19. doi: 10.1111/apel.12299

[ref54] Klug K. Selenko E. Van Hootegem A. Sverke M. De Witte H. (2024). A lead article to go deeper and broader in job insecurity research: understanding an individual perception in its social and political context. Appl. Psychol. 73, 1960–1993. doi: 10.1111/apps.12535

[ref55] Kuntz J. R. C. Malinen S. Näswall K. (2017). Employee resilience: directions for resilience development. Consult. Psychol. J. Pract. Res. 69, 223–242. doi: 10.1037/cpb0000097

[ref56] Kvasková L. Hlaďo P. Palíšek P. Šašinka V. Hirschi A. Ježek S. . (2023). A longitudinal study of relationships between vocational graduates’ career adaptability, career decision-making self-efficacy, vocational identity clarity, and life satisfaction. J. Career Assess. 31, 27–49. doi: 10.1177/10690727221084106

[ref57] Lincoln Y. S. Guba E. G. (1986). But is it rigorous? Trustworthiness and authenticity in naturalistic evaluation. New Dir. Program Eval. 1986, 73–84. doi: 10.1002/ev.1427

[ref58] Loghman S. Quinn M. Dawkins S. Woods M. Sharma S. O. Scott J. (2023). A comprehensive meta-analyses of the nomological network of psychological capital (PsyCap). J. Leadersh. Organ. Stud. 30, 108–128. doi: 10.1177/15480518221107998

[ref59] Luthans F. Avolio B. J. Avey J. B. Norman S. M. (2007). Positive psychological capital: measurement and relationship with performance and satisfaction. Pers. Psychol. 60, 541–572. doi: 10.1111/j.1744-6570.2007.00083.x

[ref60] Luthans F. Youssef-Morgan C. M. (2017). Psychological capital: an evidence-based positive approach. Annu. Rev. Organ. Psychol. Organ. Behav. 4, 339–366. doi: 10.1146/annurev-orgpsych-032516-113324

[ref61] Makarius E. E. Mukherjee D. Fox J. D. Fox A. K. (2020). Rising with the machines: a sociotechnical framework for bringing artificial intelligence into the organization. J. Bus. Res. 120, 262–273. doi: 10.1016/j.jbusres.2020.07.045

[ref62] Mayring P. (2014). “Qualitative content analysis: theoretical background and procedures,” in Approaches to Qualitative Research in Mathematics Education, eds. Bikner-Ahsbahs A. Knipping C. Presmeg N. (Berlin: Springer), 365–380.

[ref63] Mertanen K. Pashby K. Brunila K. (2020). Governing of young people “at risk” with the alliance of employability and precariousness in the EU youth policy steering. Policy Futures Educ. 18, 240–260. doi: 10.1177/1478210319838666

[ref64] Morse J. M. Barrett M. Mayan M. Olson K. Spiers J. (2002). Verification strategies for establishing reliability and validity in qualitative research. Int J Qual Methods 1, 13–22. doi: 10.1177/160940690200100202

[ref65] Näswall K. Malinen S. Kuntz J. Hodliffe M. (2019). Employee resilience: development and validation of a measure. J. Manager. Psychol. 34, 353–367. doi: 10.1108/JMP-02-2018-0102

[ref66] Newman A. Donohue R. Eva N. (2017). Psychological safety: a systematic review of the literature. Hum. Resour. Manag. Rev. 27, 521–535. doi: 10.1016/j.hrmr.2017.01.001

[ref67] Orfao G. Malo M. Á. (2023). Are active labour market policies effective for the older unemployed? A meta-evaluation. Ageing Soc. 43, 1617–1637. doi: 10.1017/S0144686X21001288

[ref68] Pansini M. Buonomo I. De Vincenzi C. Ferrara B. Benevene P. (2023). Positioning technostress in the JD-R model perspective: a systematic literature review. Healthcare 11:446. doi: 10.3390/healthcare11030446, 36767021 PMC9914396

[ref69] Parker S. K. Grote G. (2022). Automation, algorithms, and beyond: why work design matters more than ever in a digital world. Appl. Psychol. 71, 1171–1204. doi: 10.1111/apps.12241

[ref70] Rani U. Furrer M. (2021). Digital labour platforms and new forms of flexible work in developing countries: algorithmic management of work and workers. Competition. Change 25, 212–236. doi: 10.1177/1024529420905187

[ref71] Rice S. Leahy M. Klatt G. (2022). Navigating the matrix: career development and the responsibilisation of employability in Australia. Br. J. Guid. Couns. 50, 557–576. doi: 10.1080/03069885.2022.2057923

[ref72] Rudolph C. W. Lavigne K. N. Zacher H. (2017). Career adaptability: a meta-analysis of relationships with measures of adaptivity, adapting responses, and adaptation results. J. Vocat. Behav. 98, 17–34. doi: 10.1016/j.jvb.2016.09.002

[ref73] Savickas M. L. Porfeli E. J. (2012). Career adapt-abilities scale: construction, reliability, and measurement equivalence across 13 countries. J. Vocat. Behav. 80, 661–673. doi: 10.1016/j.jvb.2012.01.011

[ref74] Schaufeli W. B. Taris T. W. (2014). “A critical review of the job demands-resources model: implications for improving work and health,” in Bridging Occupational, Organizational and Public Health: A Transdisciplinary Approach, eds. Bauer G. F. Hämmig O. (Berlin: Springer), 43–68.

[ref75] Schlogl L. Weiss E. Prainsack B. (2021). Constructing the ‘future of work’: an analysis of the policy discourse. New Technol. Work Employ. 36, 307–326. doi: 10.1111/ntwe.12202

[ref76] Scholze A. Hecker A. (2024). The job demands-resources model as a theoretical lens for the bright and dark side of digitization. Comput. Hum. Behav. 155:108177. doi: 10.1016/j.chb.2024.108177

[ref77] Schreier M. (2014). “Qualitative content analysis,” in The SAGE Handbook of Qualitative Data Analysis, ed. Flick U. (Thousand Oaks, CA: SAGE), 170–183.

[ref78] Shoss M. K. (2017). Job insecurity: an integrative review and agenda for future research. J. Manag. 43, 1911–1939. doi: 10.1177/0149206317691574

[ref79] Siegel R. König C. J. Porsch L. (2021). Does electronic monitoring pay off? Influences of electronic monitoring purposes on organizational attractiveness. J. Pers. Psychol. 20, 103–113. doi: 10.1027/1866-5888/a000273

[ref80] Şimşek V. Dilekçi Ü. (2025). Occupational stress and occupational resilience as predictors of teachers' job performance. Milli Egitim Derg. 54, 1869–1904. doi: 10.37669/milliegitim.1666077

[ref81] Snyder C. R. (2002). Hope theory: rainbows in the mind. Psychol. Inq. 13, 249–275. doi: 10.1207/S15327965PLI1304_01

[ref82] Sonnentag S. Meier L. L. (2024). Gain and loss cycles revisited: what to consider when testing key assumptions of conservation of resources theory. J. Manag. Scient. Rep. 2, 154–167. doi: 10.1177/27550311241247833

[ref83] Suslovic B. Lett E. (2024). Resilience is an adverse event: a critical discussion of resilience theory in health services research and public health. Commun. Health Equity Res. Pol. 44, 339–343. doi: 10.1177/2752535X231159721, 36856261 PMC10919062

[ref84] Tracy S. J. (2010). Qualitative quality: eight “big-tent” criteria for excellent qualitative research. Qual. Inq. 16, 837–851. doi: 10.1177/1077800410383121

[ref85] van den Berg G. J. Dauth C. Homrighausen P. Stephan G. (2023). Informing employees in small and medium-sized firms about training: results of a randomized field experiment. Econ. Inq. 61, 162–178. doi: 10.1111/ecin.13111

[ref86] van Hulst M. Yanow D. (2016). From policy “frames” to “framing”: theorizing a more dynamic, political approach. Am. Rev. Public Adm. 46, 92–112. doi: 10.1177/0275074014533142

[ref87] Vignola E. F. Baron S. Abreu Plasencia E. Hussein M. Cohen N. (2023). Workers’ health under algorithmic management: emerging findings and urgent research questions. Int. J. Environ. Res. Public Health 20:1239. doi: 10.3390/ijerph20021239, 36673989 PMC9859016

[ref88] Vincent S. Lopes A. Meliou E. Özbilgin M. (2024). Relational responsibilisation and diversity management in the 21st century: the case for reframing equality regulation. Work Employ. Soc. 38, 1549–1568. doi: 10.1177/09500170231217660

[ref89] Vooren M. Haelermans C. Groot W. Maassen van den Brink H. (2019). The effectiveness of active labor market policies: a meta-analysis. J. Econ. Surv. 33, 125–149. doi: 10.1111/joes.12269

[ref90] Walczok M. Bipp T. (2025). Understanding the emergence and trajectory of job insecurity due to smart technology, artificial intelligence, robotics, and automation. Hum. Factors Ergon. Manuf. Serv. Ind. 35:e70000. doi: 10.1002/hfm.70000

[ref91] Yön Ö. Dilekçi Ü. (2026). Relationship between teachers’ emotions and their professional resilience. Trakya Egit. Derg. 16, 1522–1548. doi: 10.24315/tred.1785713

