# Supplementary material for: The Resilience Responsibility Gap: how institutions distribute the burden of adaptation under AI-driven structural disruption
Source: Front Psychol. 2026 Oct 16;17:1974636. doi: 10.3389/fpsyg.2026.1974636 (PMC13719421; doi:10.3389/fpsyg.2026.1974636)
Supplement: Supplementary file 1 [file Data_Sheet_1.docx]

**Appendix A. Corpus of analysed institutional documents (D01–D18)**

*Note.* Documents are cited in the text by identifier. Publication years follow the year stated on the publication itself; where a corpus filename reflects a project registration year rather than the publication year, the published year is used (see D16). D09 is published as a European Commission Joint Research Centre working paper produced in collaboration with EU-OSHA and Eurofound. D10 is an ILO briefing note submitted to the Senate of Canada; no ILO-hosted version was identified, so the submission is cited as the primary source.

**D01**: OECD (2023). *OECD Employment Outlook 2023: Artificial Intelligence and the Labour Market*. Report. Focus: AI labour-market transformation; worker/employer perceptions; skills; job quality. https://www.oecd.org/content/dam/oecd/en/publications/reports/2023/07/oecd-employment-outlook-2023_904bcef3/08785bba-en.pdf

**D02**: OECD (2024). *Artificial Intelligence and the Changing Demand for Skills in the Labour Market*. Working paper. Focus: Skill obsolescence and changing task/skill demand. https://www.oecd.org/content/dam/oecd/en/publications/reports/2024/04/artificial-intelligence-and-the-changing-demand-for-skills-in-the-labour-market_861a23ea/88684e36-en.pdf

**D03**: OECD (2025). *Bridging the AI Skills Gap: Is Training Keeping Up?*. Policy brief. Focus: Reskilling responsibility; accessibility and inclusivity of training. https://www.oecd.org/content/dam/oecd/en/publications/reports/2025/04/bridging-the-ai-skills-gap_b43c7c4a/66d0702e-en.pdf

**D04**: OECD (2026). *AI and Skills: What We Know So Far*. Policy brief. Focus: Shared responsibility among employers, workers and governments. https://www.oecd.org/content/dam/oecd/en/publications/reports/2026/06/ai-and-skills_8dbed5fe/f843b352-en.pdf

**D05:** OECD (2026). *Skills in the AI Age* (OECD Artificial Intelligence Papers, No. 60). Working paper. Focus: Structural skills gaps, adaptation capacity and unequal exposure. https://www.oecd.org/content/dam/oecd/en/publications/reports/2026/07/skills-in-the-ai-age_e8d8c1e6/972bd15e-en.pdf

**D06**: Eurofound (2024). *Human–robot interaction: What changes in the workplace?*. Report. Focus: Organisational choices, worker involvement, training, autonomy and psychosocial risk. https://assets.eurofound.europa.eu/f/279033/dc14f762ca/ef23010en.pdf

**D07**: Eurofound (2021). *Digitisation in the workplace*. Report. Focus: Employee involvement, social dialogue, work organisation and job quality. https://assets.eurofound.europa.eu/f/279033/5f7b7aa5eb/ef21001en.pdf

**D08**: Eurofound (2022). *Ethics in the digital workplace*. Report. Focus: Algorithmic management, monitoring, rights, job quality and policy responsibility. https://assets.eurofound.europa.eu/f/279033/387c3d1b6f/ef22038en.pdf

**D09: Urzì Brancati, M. C., Curtarelli, M., Riso, S., & Baiocco, S.** (2022). *How digital technology is reshaping the art of management* (JRC Working Papers Series on Labour, Education and Technology, No. 2022/05). European Commission, Joint Research Centre. Working paper. Focus: Data-driven management, autonomy, surveillance, wellbeing and organisational mitigation. https://joint-research-centre.ec.europa.eu/system/files/2022-11/JRC130808_How%20digital%20technology%20is%20reshaping%20the%20art%20of%20management_LET%20WP%2005-22_0.pdf

**D10: International Labour Organization (2026). ILO Briefing Note:** *AI and the World of Work [Brief submitted to the Standing Senate Committee on Human Rights, Senate of Canada].* Policy brief. Focus: AI-driven structural change; skills, job quality, inequality, workers’ rights, social protection and social dialogue. https://sencanada.ca/Content/Sen/Committee/451/RIDR/briefs/InternationalLabourOrganization_e.pdf

**D11**: OECD (2026). *Recent policy developments on AI in the labour market*. Review. Focus: Current policy allocation of responsibility and worker protections. https://www.oecd.org/content/dam/oecd/en/publications/reports/2026/07/recent-policy-developments-on-ai-in-the-labour-market_52d4afe2/c0ffced7-en.pdf

**D12**: EU-OSHA (2025). *OSH Pulse 2025: Occupational safety and health in the era of climate and digital change*. Report. Focus: Psychosocial risk, job insecurity, algorithmic management and workplace prevention. https://osha.europa.eu/sites/default/files/documents/OSH-pulse-2025-climate-digital-change_EN.pdf

**D13**: OECD (2025). *AI Adoption by Small and Medium-sized Enterprises*. Report. Focus: Organisational capability and barriers to AI adoption; training and skills context. https://www.oecd.org/content/dam/oecd/en/publications/reports/2025/12/ai-adoption-by-small-and-medium-sized-enterprises_9c48eae6/426399c1-en.pdf

**D14**: OECD (2025). *Artificial Intelligence and the Labour Market in Korea*. Report. Focus: Cross-national policy and organisational adaptation case. https://www.oecd.org/content/dam/oecd/en/publications/reports/2025/10/artificial-intelligence-and-the-labour-market-in-korea_af668423/68ab1a5a-en.pdf

**D15**: OECD (2025). *Artificial Intelligence and the Labour Market in Japan*. Report. Focus: Cross-national case; training, worker attitudes, psychosocial and organisational dimensions. https://www.oecd.org/content/dam/oecd/en/publications/reports/2025/11/artificial-intelligence-and-the-labour-market-in-japan_a67a343c/b825563e-en.pdf

**D16**: Eurofound (2024). *Self-employment in the EU: Job quality and developments in social protection*. Report. Focus: Precarity/platform work and unequal access to resilience resources. https://assets.eurofound.europa.eu/f/279033/31e15ac4b3/ef23004en.pdf

**D17**: EU-OSHA (2023). *Safe and healthy work in the digital age 2023–2025: Campaign guide*. Guideline. Focus: Digitalisation risks, organisational prevention and worker participation. https://healthy-workplaces.osha.europa.eu/sites/hwc/files/hwc/publication/HWC-23-25_Campaign_Guide_en.pdf

**D18**: Eurofound (2025). *Implications of AI for work, employment and social dialogue: Literature review*. Working paper. Focus: Work organisation, job quality, worker voice, social dialogue, skills, psychological contract and distributional consequences of AI. https://www.eurofound.europa.eu/en/publications/all/implications-of-ai-for-work-employment-and-social-dialogue-literature-review
